# Repetitive genomic insertion of gene-sized dsDNAs by targeting the promoter region of a counter-selectable marker

**DOI:** 10.1038/srep08712

**Published:** 2015-03-04

**Authors:** Jaehwan Jeong, Han Na Seo, Yu Kyung Jung, Jeewon Lee, Gyuri Ryu, Wookjae Lee, Euijin Kwon, Keunsoo Ryoo, Jungyeon Kim, Hwa-Young Cho, Kwang Myung Cho, Jin Hwan Park, Duhee Bang

**Affiliations:** 1 Department of Chemistry, Yonsei University, 50 Yonsei-ro, Seodaemun-gu, Seoul, 120-749, Korea; 2 Samsung Advanced Institute of Technology, 130 Samsung-ro, Yeongtong-gu, Suwon-si, Gyeonggi-do, 443-803, Korea; 3 Department of Chemistry, University College London, 20 Gordon Street, London, WC1H 0AJ, U.K

## Abstract

Genome engineering can be used to produce bacterial strains with a wide range of desired phenotypes. However, the incorporation of gene-sized DNA fragments is often challenging due to the intricacy of the procedure, off-target effects, and low insertion efficiency. Here we report a genome engineering method enabling the continuous incorporation of gene-sized double-stranded DNAs (dsDNAs) into the *Escherichia coli* genome. DNA substrates are inserted without introducing additional marker genes, by synchronously turning an endogenous counter-selectable marker gene ON and OFF. To accomplish this, we utilized λ Red protein-mediated recombination to insert dsDNAs within the promoter region of a counter-selectable marker gene, *tolC*. By repeatedly switching the marker gene ON and OFF, a number of desired gene-sized dsDNAs can be inserted consecutively. With this method, we successfully inserted approximately 13 kb gene clusters to generate engineered *E. coli* strains producing 1,4-butanediol (1,4-BDO).

Bacterial genome engineering involves using techniques such as gene insertion, deletion, and substitution to manipulate genomic DNA and has enabled the production of strains with a wide range of applications. These include the targeted engineering of biosynthetic and evolutionary pathways, analysis of network systems, phenotypic behavioral studies, and biological discovery^1^. The broad applicability of engineered bacterial strains has highlighted the increased need for efficient genome engineering methods, and the insertion of protein-coding genes into genomes remains critical for obtaining strains with new or significantly improved functions^2^. In particular, the development of strains producing industrial biochemicals through metabolic engineering is dependent upon the introduction of foreign protein-coding genes in order to achieve functions that did not previously exist in a particular bacterial organism^3^,^4^,^5^. To construct strains with the desired genotype, plasmids containing the foreign genes of interest are typically utilized. However, plasmid-based strains are not sufficient for industrial scale biochemical production purposes due to yield variation resulting from copy number variability^6^ and instability of the plasmid in large-scale culture^7^.

Genome-based strain development can be achieved by assembly and integration of multi-genes that consist of the desired biosynthetic pathway. For multi-gene assembly, *B. subtilis* and *S. cerevisiae* are ideal host strains, because they incorporate exogenous genes properly and assemble DNA fragments in a highly efficient manner^8^,^9^. Moreover, the assembled product is stably maintained in their genome. However, when we need to use *E. coli* as a host strain of the assembled biosynthetic pathway, additional transfer of the assembled product into *E. coli* is required. For example, the carotenoid biosynthetic pathway was successfully assembled from several gene pieces in *B. subtilis*. However, it required an additional transfer into *E. coli* before it could be assayed^10^. The inconvenience of the transfer step may depreciate the advantages of this assembly method.

Efficient integration of large DNA constructs into *E. coli* genome could potentially be attained using phage-derived integrase. By utilizing the attachment sites *attP* and *attB*, which are λ integrase recognition sites, DNA fragments can be integrated into the host genome at the desired target site^11^,^12^. However, the attachment sites should be integrated into the intended genome location prior to introducing DNA fragments. This method produces a ‘scar,' i.e., attachments site sequences in the host genome. Utilizing the homing endonuclease I-SceI is another approach for DNA fragment integration into a genome. With introduction of the ‘landing pad' sequence to target site flanked by I-SceI site, DNA fragment can be incorporated into genome via induction of I-SceI and λ Red proteins^13^. Although a 7-kb DNA fragment could potentially be integrated to the *E. coli* genome using this method, it is restricted by the residual ‘scar' on the genome and the need to incorporate the ‘landing pad' sequence prior to DNA fragment introduction.

For efficient, scarless genomic integration, λ Red recombination-based genome engineering has been frequently used^14^. However, due to the requirement that the DNA fragment must be incorporated as an Okazaki fragment, λ Red recombination alone is limited when inserting longer DNA strands^13^,^15^. Although a 7.3-kb DNA fragment can be incorporated into the *E. coli* genome through Knock-in/Knock-out (KIKO) vectors, this system requires long homology arms (~500 bp) and a selection marker gene inside the DNA fragment to select the properly dsDNA fragment integrated *E. coli*^16^. These two features increase DNA fragment length resulting in decreased recombination efficiency.

Here, we describe an integration method that enables continuous incorporation of gene-sized dsDNA fragments into the *Escherichia coli* genome. Importantly, fragments are inserted without introducing additional marker genes via manipulation of the promoter region of the counter-selectable marker gene, *tolC*^17^. Using this insertion method with λ Red recombination, we were able to engineer wild-type *E. coli* to contain exogenous genes that produce 1,4-butanediol (1,4-BDO) which is widely used for industrial purposes such as organic solvent and material of manufacturing various plastics. Further, we gained insight into the possible industrial utility of the 1,4-BDO-producing strains by phenotypically comparing genome-integrated strains with plasmid-based expression strains.

## Results

### General scheme for scarless genomic insertion of multiple gene-sized dsDNAs

**Figure 1:**
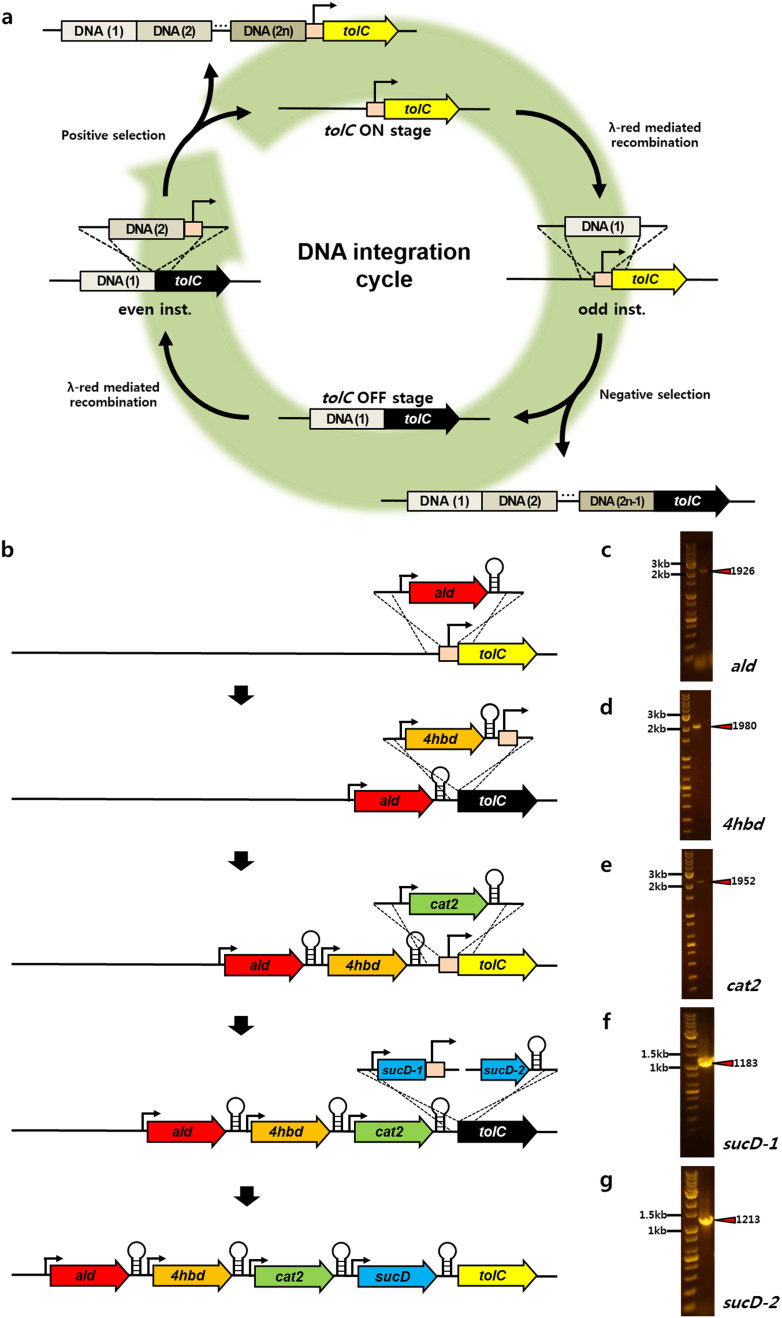
A schematic representation of repetitive genomic insertion of dsDNA substrates. (a) A schematic representation of the sequential 1,4-BDO gene cluster insertion using the counter-selectable marker, *tolC*. Each gene was inserted separately next to the promoter region of *tolC* by λ Red-mediated homologous recombination. Insertion of *sucD* was operated with two divided substrates to overcome the reduced selection efficiency. (b) Genomic insertion of the 1,4-BDO biosynthetic pathway gene cluster and agarose gel electrophoresis to confirm the insertion of (c) *ald*, (d) *4hbd*, (e) *cat2*, (f) *sucD*-1, and (g) *sucD*-2.

To enable the targeted genomic insertion of foreign genes with λ Red recombination, dsDNA substrates were generated by PCR to contain the gene of interest and 50 bp of sequence homologous to the target insertion site on either end (Fig. 1a). In each case, the λ Red proteins were induced by 15 min of incubation at 42°C^14^, and the prepared substrates were introduced to *E. coli* via electroporation. For the continuous introduction of multiple genes, odd-ordered insertions were designed to target the promoter region of *tolC*, resulting in its deletion. With the proper insertion of the substrate, the transcription of the *tolC* gene cannot occur due to the absence of its promoter, and cells will lack the outer membrane protein, TolC. Therefore, only the cells with a dsDNA substrate-inserted genotype, which is in OFF stage of TolC, can be negatively selected in media containing Colicin E1, as its influx and ability to target the cytoplasmic membrane are blocked in these cells^17^.

For even-ordered insertions, which are obtained by positive selection, dsDNA substrates were designed to contain the intact *tolC* promoter, thereby reconstituting gene expression. In this case, substrates that are properly inserted into the clone alter the status of TolC from OFF to ON and express functional TolC protein. Clones are positively selected with sodium dodecyl sulfate (SDS), which can penetrate the cell membrane resulting in cell death. Cells expressing TolC, via gene insertion and promoter recovery, however, can efflux the toxic SDS out of cell and survive^17^. Therefore, utilizing multiple odd- and even-ordered insertions, which turn TolC OFF and ON, respectively, whole genes or gene fragments can be sequentially integrated into the *E. coli* genome and screened by repetitive counter selections.

### Genomic insertion of 1,4-BDO biosynthetic pathway genes

**Figure 2:**
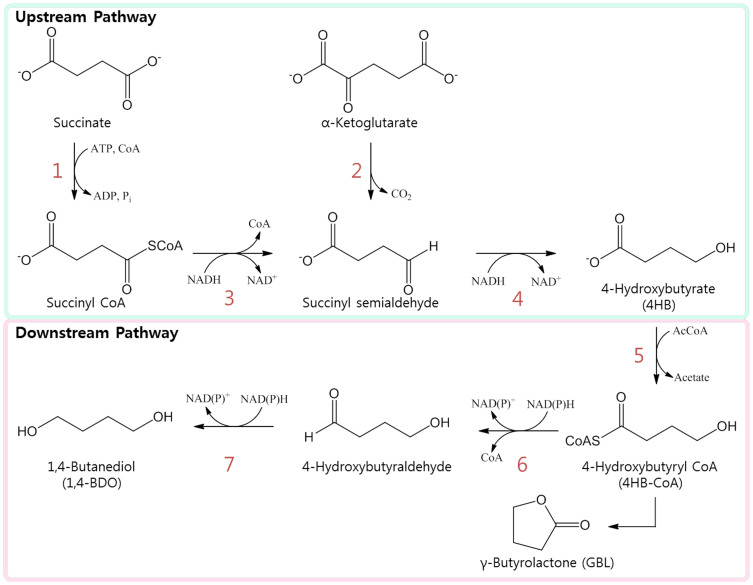
The 1,4-BDO biosynthetic pathway. The metabolic pathway for 1,4-butanediol (1,4-BDO) biosynthesis in YSB20 consists of: **1**. succinyl-CoA synthetase (SCS), endogenous *sucCD* from *E. coli*; **3**. succinate semialdehyde dehydrogenase (SSADH), *sucD* from *Porphyromonas gingivalis*; **4**. 4-hydroxybutyrate dehydrogenase (4HBD), *4hbd* from *P. gingivalis*; **5**. 4HB-CoA transferase (4HB-CoAT), *cat2* from *P. gingivalis*; **6**. 4-hydroxybutyryl-CoA reductase, *ald* from *Clostridium beijerinckii*; and **7**. alcohol dehydrogenase, endogenous *adh* from *E. coli*. The engineered 1,4-BDO biosynthetic pathway of YSB27 and W029-11 consists of **1**. succinyl-CoA synthetase (SCS), endogenous *sucCD* from *E. coli*; **2**. α-ketoglutarate decarboxylase, *kgd* from *Corynebacterium glutamicum*; **3**. succinate semialdehyde dehydrogenase (SSADH), *sucD* from *P. gingivalis*; **4**. 4-hydroxybutyrate dehydrogenase (4HBD), *4hbd* from *P. gingivalis*; **5**. 4HB-CoA transferase (4HB-CoAT), *cat2* from *P. gingivalis*; **6**. butyraldehyde dehydrogenase, *bld_M_* from *Clostridium saccharoperbutylacetonicum*; and **7**. alcohol dehydrogenase, endogenous *adh* from *E. coli*.

In order to engineer *E. coli* that efficiently produce 1,4-BDO, we deleted *mdh*, *ldhA*, *arcA*, *adhE*, and *pflB* by truncation from the parent strain, EcNR2, to inhibit the major 1,4-BDO fermentation pathway and to enhance the flux of the 1,4-BDO biosynthetic pathway (Supplementary Table 1). We then introduced a *gltA* R164L mutation and substituted the endogenous *lpdA* gene with *Klebsiella pneumoniae lpdA* E354K, to generate the strain, YSB11 (Table 1). It has previously been shown that the introduction of four foreign genes into *E. coli* (*sucD*, *4hbd*, and *cat2* from *Porphyromonas gingivalis*, and *ald* from *Clostridium beijerinckii*, Supplementary Table 2) enabled 1,4-BDO production from succinate^18^, an intermediate of the TCA cycle. *E. coli*'s native succinyl-CoA synthetase (SCS, encoded by *sucCD*) converts succinate into succinyl-CoA. This intermediate is then modified by reactions with the CoA-dependent succinate-semialdehyde dehydrogenase (SSADH, encoded by *sucD*) and 4-hydroxybutyrate dehydrogenase (4HBD, encoded by *4hbd*), to form 4-hydroxybutyrate (4HB). Transfer of CoA to 4HB is mediated by 4-hydroxybutyrate-CoA transferase (4HB-CoAT, encoded by *cat2*) and generates 4HB-CoA. Final reductions of 4HB-CoA by 4-hydroxybutyryl-CoA reductase (encoded by *ald*) and endogenous alcohol dehydrogenases (encoded by the *adh* genes) from *E. coli* produce 1,4-BDO (Fig. 2).

Foreign genes were inserted into YSB11 in the following order: *ald*, *4hbd*, *cat2*, and *sucD* (Fig. 1b and Supplementary Table 2). Each individual gene was flanked by unique promoter^19^ and terminator^20^ sequences to prevent them from looping out via homologous recombination (Supplementary Tables 4 and 5). The first insertion, *ald* (1,726 bp), was designed to disrupt the 200 bp promoter region and eliminate transcription of *tolC*. Through negative selection with Colicin E1, we were able to isolate clones containing the correctly targeted insertion (Fig. 1c). The next insertion, *4hbd* (1,528 bp), was introduced between *ald* and *tolC*. This substrate was designed to reconstitute the *tolC* promoter, such that the gene will only be transcribed if the *4hbd* fragment is inserted properly. We then used SDS-mediated positive selection to identify *4hbd*-inserted clones (Fig. 1d). In the third round of targeting, the *cat2* (1,626 bp) gene was inserted in the same manner as *ald*; that is, the substrate was designed to disrupt the *tolC* promoter region, which was previously inserted with *4hbd*, and clones were screened by another round of negative selection (Fig. 1e). The final gene, *sucD* (1,782 bp), was inserted between *cat2* and *tolC* as two divided substrates (999 bp, 883 bp). By positive selection followed by negative selection, we were then able to isolate the correct clones, which contained a complete genome-integrated 1,4-BDO biosynthetic pathway and generate the *E. coli* strain YSB20 (Fig. 1b, f and g, Table 1).

### Reduced efficiency of DNA fragment insertion with repetitive use of *tolC* counter selection

During the construction of YSB20, it was noted that the repeated use of *tolC* counter selection with λ Red recombination showed a tendency for reduced selection efficiency. The initial insertion by negative selection and secondary insertion by positive selection were obtained with efficiencies of 69.7% and 98.5%, respectively. However, the efficiencies of the third and fourth gene insertions through negative selection, followed by positive selection, decreased dramatically to 35.7% and 0%, respectively.

**Figure 3:**
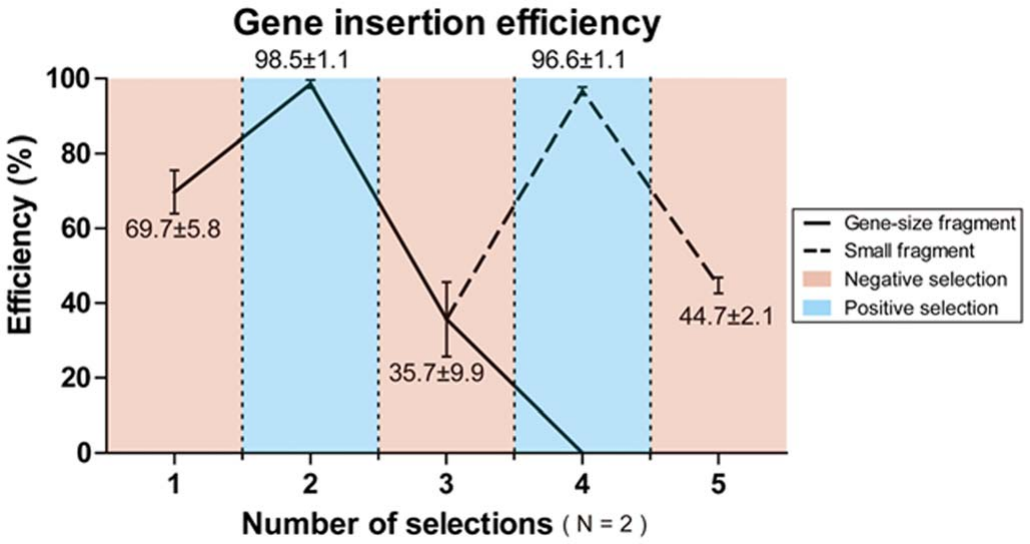
Efficiency of genomic insertion followed by counter selection during construction of YSB20. For the *ald* insertion to *tolC* promoter region, negative selection of *tolC* was conducted and its efficiency showed 69.7%. Selection efficiency of sequentially introduced *4hbd*, *cat2*, and *sucD* showed 98.5%, 35.7% and 0%, respectively. The last substrate, *sucD* was divided into two dsDNA substrates for its insertion, *sucD*-1 and *sucD*-2. The insertion efficiencies of *sucD*-1 and *sucD*-2 were recovered to 96.6% and 44.7%, respectively. Each test was done in duplicate, and results are presented as mean ± s.d.

We hypothesized that shorter dsDNA fragments could be converted into ssDNA more easily due to the 5′ exonuclease activity of the λ Exo protein^15^. The easy generation of ssDNA might increase the probability of mutation, thereby increasing the frequency with which mutant clones would be detected. In order to test this hypothesis, we reduced the length of the *sucD* dsDNA substrate, which was initially 1,782 bp, by dividing it into two parts. The insertion efficiency of the first 999 bp fragment, containing the *tolC* promoter together with the 5′ part of the *sucD* gene (*sucD*-1), was enhanced to 96.6%. The remaining portion of the *sucD* gene, *sucD-*2, which is another 883 bp, was introduced by disrupting *tolC* promoter, with an insertion efficiency of 44.7% (Fig. 3).

Repeated use of λ Red recombination for the insertion of DNA fragments has been reported to increase the number of off-target mutation events in other parts of the genome. Especially, accumulation of unwanted mutations in *tolQRA*, which is important for Colicin E1 uptake to cytosol by formation of membrane spanning protein complex^21^,^22^, is resulting in a decreased selection efficiency^23^. However, we were able to successfully incorporate dsDNAs into the genome with recovered efficiency by reducing the length of the dsDNA substrates.

### Genomic insertion of the engineered 1,4-BDO biosynthetic pathway

Although the complete 1,4-BDO biosynthetic pathway, comprised of *sucD*, *4hbd*, *cat2*, and *ald*, was successfully inserted into the genome of *E. coli* strain YSB11, the resulting YSB20 strain synthesized only negligible amounts of 1,4-BDO (data not shown). Thus, we attempted to modify the 1,4-BDO biosynthetic pathway to increase 1,4-BDO production. To accomplish this, additional copies of the *E. coli* *sucCD* genes, which are responsible for the conversion of succinate into succinyl-CoA, were inserted to enhance the initial step of 1,4-BDO synthesis pathway. We then integrated the *kgd* (NCgl1084) gene from *Corynebacterium glutamicum* to mediate the conversion of α-ketoglutarate into succinyl semialdehyde, further increasing the metabolic flux for synthesis of 4HB, a necessary intermediate in the 1,4-BDO pathway. Finally, the aldehyde dehydrogenation step, which is considered to be limiting for the conversion of 4HB to 1,4-BDO, was enhanced by replacing *ald* with the mutated *bld* (*bld* M227L, L273I) from *Clostridium saccharoperbutylacetonicum*^24^, as this has been shown to encode a protein with higher activity than that encoded by the *C. beijerinckii ald* gene (data not shown).

For the further investigation of metabolic flux based on the copy number variation of the genes, we divided the engineered 1,4-BDO pathway into two parts based on the upstream and downstream of 4HB. The upstream pathway, comprised of *sucCD*, *sucD*, *4hbd*, and *kgd*, was placed under the control of the IPTG-inducible *tac* promoter and *rrnB* transcription terminator. Expression of downstream pathway genes, *bld_M_* and *cat2*, was put under the control of the IPTG-inducible *trc* promoter and the *rrnB* transcription terminator (Fig. 2, Supplementary Table 3). Both upstream and downstream pathways were cloned into plasmids to generate pTac15K sucCD-sucD-4hbd-kgd and pTrc99A bld_M_-cat2, respectively. Due to the dramatic decrease in λ Red recombination efficiency when the DNA fragment length exceeds 2 kb^13^, DNA fragments, approximately 2 kb in size, containing the 1,4-BDO biosynthetic genes and their regulatory elements were prepared using PCR amplification of the upstream and downstream pathway plasmids and introduced into the *E. coli* genome by targeting the *tolC* promoter region. We then integrated the engineered 1,4-BDO biosynthetic pathway into the *E. coli* strain YSB11 using λ Red recombination and *tolC* counter selection, to generate YSB25 (containing only the upstream genes) and YSB27 (containing the entire pathway) (Table 1, Supplementary Note 1, Supplementary Fig. 3). However, YSB25 showed no detectable amounts of 4HB and YSB27 produced 10.00 mg/L 1,4-BDO.

In order to improve yield of 1,4-BDO production, we integrated the entire pathway into a derivative of the *E. coli* strain W, which has been shown to be suitable for industrial bioprocesses due to its ability to use sucrose as carbon source^25^. We utilized the previously reported W023 strain^24^, an *E. coli* W derivative with the modifications described above: deletions of *mdh, ldhA*, *arcA*, *adhE*, and *pflB*, as well as the *gltA* R164L mutation and a substitution of *lpdA* with the homologue from *K. pneumoniae* containing E354K.

The strain W029 was prepared by further engineering W023, as follows: i) major back-fluxes interfering with 1,4-BDO synthesis were blocked by knocking out *gabD*, *sad*, and *puuC* genes; ii) acetyl-CoA synthetase (ACS, encoded by *acs*), which catalyzes conversion of acetate to acetyl-CoA, was overexpressed to increase pools of intracellular acetyl-CoA, which is required for the Cat2 reaction during 1,4-BDO synthesis; iii) phosphoenolpyruvate carboxylase (PEPC, encoded by *ppc*), an anaplerotic enzyme of TCA cycle, was also overexpressed to further enhance the TCA cycle, thereby increasing glycolytic flux towards 1,4-BDO production (Supplementary Note 2). Due to the lack of genomic λ Red recombination system in W029, we also transformed W029 with pEKEx2-Red and knocked-out *mutS* using the *cat* cassette^26^.

To integrate the 1,4-BDO upstream pathway, we attempted rounds of insertions with smaller DNA fragments: 1,660 bp, 1,660 bp, 830 bp, 696 bp, and 765 bp. With the repeated use of *tolC* counter selection utilizing Colicin E1 and SDS, we successfully inserted the entire 1,4-BDO upstream pathway, generating the strain W029-7. The downstream pathway was then introduced with 1,017 bp, 1,163 bp, and 1,043 bp substrates by targeting the *tolC* promoter region with successive rounds of counter selection. The 1,017 bp and 1,163 bp substrates were introduced successfully, but the last fragment, 1,043 bp, was unable to be inserted due to the reduced efficiency of *tolC* counter selection. As shown in previous strain constructions, we divided the 1,043 bp substrate into 503 bp and 540 bp fragments. The 503 bp fragment was successfully inserted and recovered by positive selection, whereas insertion clones containing the 540 bp DNA fragment were not found through negative selection. In previous strain constructions, we had noted that positive selection showed relatively better insertion efficiency than negative selection. Therefore, we first removed the *tolC* promoter that had been introduced with the 503 bp fragment in the previous step. We then inserted the 540 bp fragment using a substrate that reconstitutes the *tolC* promoter. Using this strategy, we obtained the strain W029-11, which contains the entire BDO biosynthetic pathway integrated into the genome of W029 (Table 1, Supplementary Fig. 2).

### Production level of 1,4-BDO

We next compared production levels of 4HB and 1,4-BDO in our engineered strains. Due to the fact that the copy number of genes involved in a biosynthetic pathway can affect the flux of the pathway^27^,^28^, we evaluated strains having various copies of the 1,4-BDO biosynthetic genes. TolC is a transmembrane channel protein and its absence can affect the efflux of cytoplasmic 1,4-BDO into the culture media. In strain W029-7, which contains the upstream pathway genes, the *tolC* promoter was removed during the last DNA fragment insertion by negative selection. Therefore, to recover the expression of TolC in this strain, wild-type *tolC* promoter was introduced in front of the *tolC* ORF. W029-12 was then prepared by transformation of W029-7 with pTrc99a bld_M_-cat2, which encodes the downstream pathway genes, in order to determine copy number effect on the downstream pathway. Likewise, W029-13 was prepared by transformation of W029 with both pTac15k sucCD-sucD-4hbd-kgd and pTrc99a bld_M_-cat2. Each strain was induced with 0.25 mM IPTG and fermented for 48 hr under anaerobic conditions to produce 4HB, γ-butyrolactone (GBL, a lactonized form of 4HB), and 1,4-BDO.

In W029-7, which contains only the upstream pathway genes in single copy, 4HB was produced at a concentration of 773 mg/L. Comparatively, production of 4HB in W029-11, which contains the entire pathway in single copy, was higher than that observed in W029-7 by 247 mg/L; this strain also produced 33 mg/L of 1,4-BDO. The elevated production of 4HB in W029-11 indicates that the existence of downstream pathway, even the single copy integrated into the genome, could increase the flux of whole biosynthetic pathway, resulting in increased production of upstream pathway intermediates. However, W029-11 also accumulated 426 mg/L of GBL, which is side product from 4HB. This GBL accumulation demonstrates that one copy of downstream pathway genes cannot provide enough flux to produce significant amounts of 1,4-BDO.

Conversely, W029-12, which has the downstream pathway encoded on a multi-copy plasmid, produced 1.07 g/L of 1,4-BDO, 32-fold higher than that observed in W029-11. This suggests that the multiple copies of downstream pathway in W029-12 can increase the flux of whole biosynthetic pathway, while producing 400 mg/L of 4HB which is lower than W029-11. In addition, GBL accumulation in W029-12 was 13-fold less than that in W029-11, further demonstrating that high expression of the downstream pathway is essential for overall 1,4-BDO biosynthesis. W029-13, which has whole biosynthetic pathway expressed from plasmids, produced 2.6-fold more 1,4-BDO than W029-12. This indicates that multiple copy or enhanced expression of the whole 1,4-BDO pathway is critical for improving 1,4-BDO production in W029-11 (Table 2).

## Discussion

Our genome engineering method, which is based on manipulating the promoter region of an endogenous counter-selectable marker gene, has demonstrated marked improvements over current methods. Notably, we were able to increase insertion efficiency using dsDNA substrates of reduced length by targeting the endogenous selection marker gene *tolC* rather than incorporating an exogenous marker gene, while maintaining selection pressure to screen the desired clones. The insertion efficiency of dsDNA substrates decreases as the fragment length increases in the λ Red recombination system. By using an endogenous counter-selectable marker gene, we minimized the necessary dsDNA substrate length and, therefore, increased insertion efficiency.

The increased recombination efficiency may provide a platform to screen multiple gene variants in an efficient manner. For example, with high recombination efficiency, genomic integration of multiple gene variants is possible, and this may enable combinatorial biosynthetic pathway construction and optimization at a genomic level. To show the possibility of introducing multiple variants into genome, we introduced eight random nucleotides attached *ald* to upstream of *tolC*, and negatively selected for *tolC*. We picked eight clones, PCR amplified the target genomic locus, and Sanger sequenced. We found that all the eight clones had different sequences (Supplementary Fig. 4).

For iterative genomic DNA fragment integration, previous genome engineering methods required a new selection marker at every integration step. Alternatively, they would have to remove the previously introduced marker gene before proceeding to the next integration step with same marker gene. In our method, the initial introduction of the ‘landing pad' counter-selectable marker gene allows subsequent iterative DNA fragment integration using counter-selection. In addition, the ‘landing pad' counter-selectable marker gene can be removed easily between integration iterations using negative selection. The easy ‘landing pad' removal makes this method ‘scarless', eliminating the need for additional genomic engineering procedures. Similar to removal, the counter-selectable marker gene can easily be introduced at intended site using positive selection. For example, we removed the *thyA* gene from its original genomic site and inserted it at the desired locus (*P_tolC_*) using its counter-selection. Thus, our genomic insertion method is not limited to the fixed genomic locations of the endogenous counter-selectable marker genes (Supplementary Fig. 1).

Iterative gene insertion could also be performed using other counter-selectable markers such as *thyA*^29^, *galK*^30^, *ccdA*/*ccdB*^31^, and *rpsL*-*neo*^32^. For repeated DNA fragment insertion by λ Red recombination, the selection sustainability of these markers should be considered. Counter-selectable marker genes often require counter-selection condition titration for each experiment. We initially used *thyA* in our research; however, after the second round of gene insertion, the selection efficiency with *thyA* decreased dramatically. Consequently, we could not proceed with this method (Supplementary Note 3, Supplementary Fig. 1). In fact, when integrating the 1,4-BDO biosynthetic pathway using counter selection with *tolC*, we encountered the same problem. To overcome this, we used reduced size DNA substrates, which enhanced λ Red recombination efficiency and increased *tolC* selection efficiency. We note that there are other efforts to enhance the counter-selection efficiency. Gregg *et al*. reported that reduced *tolC* counter-selection sensitivity is caused by off-target mutations in *tolQRA*, which are accumulated due to repeated use of λ Red recombination. They overcame the problem of the reduced sensitivity by using negative selection with Colicin E1 and vancomycin^23^. Furthermore, Wang *et al*. reported that the sensitivity of counter-selectable marker can be improved by omitting Exo when the recombination process is dependent on ssDNA. The Exo-free λ Red recombination system utilizing counter-selectable *ccdA*/*ccdB* increased the effectiveness of the counter-selectable marker gene^31^.

When creating strains for industrial use, biosynthetic pathways should be integrated into the genome to avoid problems due to variation in plasmid copy number and plasmid maintenance^2^. In this study, we were able to simplify the construction of genome-integrated strains and therefore accelerate the process of creating strains for use on an industrial level. However, we found that the W029-11, which contains the biosynthetic genes integrated in single copy, produced 32-fold less 1,4-BDO than W029-12, which expressed the downstream genes from multi-copy plasmids. This difference in 1,4-BDO production could be attributed to the increased copy number of the downstream pathway genes in the plasmid-containing strain. Therefore, additional engineering should concentrate on enhancing the downstream pathway with techniques such as promoter engineering, ribosome binding site (RBS) tuning, protein engineering, and multi-copy integration to the genome. We also observed that 1,4-BDO production from W029-13, which contains the entire pathway expressed from multi-copy plasmids, was 2.79 g/L, 2.6-fold higher than that produced from W029-12, demonstrating that copy number increase of the upstream pathway also can affect the 1,4-BDO biosynthesis. However, because W029-13 showed higher accumulation of GBL (8.9-folds) than W029-12, the main issue limiting flux is downstream pathway activity.

In summary, we have demonstrated that manipulation of the promoter of an endogenous counter-selectable marker gene, *tolC*, allowed the scarless insertion of multiple genes with a high selection efficiency for genome engineering. In principle, using this technology, any gene cluster could be inserted into any strain of *E. coli*, as long as a counter-selectable marker gene is included in the insertion. We are anticipating that our genomic integration method using counter-selectable markers will be widely applicable to biotechnology and industrial applications.

## Methods

### Strains

*E. coli* EcNR2 (MG1655 Δ*bioA*/*bioB* < λ-prophage, Δ*mutS* < *ampR*) was generously provided by Harris Wang. This was modified for 1,4-BDO production by truncation knock-out of *ldhA*, *mdh*, *arcA*, *adhE*, and *pflB* (oligos used for truncation knock-out are listed in Supplementary Table 1) followed by substitution of R164L in *gltA* via electroporation^18^,^33^,^34^. We then substituted the wild-type *lpdA* gene with *lpdA* E354K mutant from *K. pneumoniae*, to generate YSB11^18^,^35^,^36^. *E. coli* W023 (Δ*ldhA*, Δ*pflB*, Δ*adhE*, Δ*lpdA*::*K. lpd* E354K, Δ*mdh*, Δ*arcA*, *gltA* R164L), a derivative of strain W, was described in a previous report^18^,^24^. The development of strain W029, the precursor for insertion of the 1,4-BDO biosynthetic pathway is described in Supplementary Note 2.

### Design and preparation of DNA substrates for genome engineering

For the construction of YSB11, each DNA substrate was designed to contain one gene, as well as its promoter and terminator (promoter and terminator sequences are listed in Supplementary Tables 4 and 5). Promoter^19^ and terminator^20^ sequences were joined to each gene by polymerase chain reaction (PCR), and 200 bp of homology to the insertion site was also added at both ends of the DNA fragment by PCR. To generate DNA substrates encoding the engineered 1,4-BDO biosynthetic pathway, each DNA substrate was designed to contain approximately 0.5 to 2 kb of DNA sequence together with 50 bp homology to the insertion site on either end. DNA substrates to be introduced to the target site by positive selection were also designed to contain the *tolC* promoter (oligos used for DNA fragment constructions are listed in Supplementary Tables 6, 7, and 8).

### Gene synthesis and cloning

1,4-BDO biosynthetic pathway genes (*ald, 4hbd, cat2, and sucD*; sequences are listed in Supplementary Table 2) for construction of YSB20 were synthesized using assembly PCR with 80 nt oligos. Sense oligos and non-sense oligos were overlapped by 40 nt. Assembly PCR was conducted in 20 μl reactions, containing 10 μl KAPA Hifi PCR kit (Kapa Biosystems, USA) reaction mix, 1 μl each of 10 μM forward and reverse primers, and 1 μl template, with the following conditions: Step 1 - 3 min at 95°C; Step 2 - 30 sec at 95°C; Step 3 - 30 sec at 60°C; Step 4 - 30 sec per 500 bp at 72°C; Step 5 - repeat steps 2 - 4 34 times; Step 6 - 10 min at 72°C. Assembled gene products were analyzed by agarose gel electrophoresis, and those with the appropriate size were excised, gel purified using QIAquick Gel Extraction kit (Qiagen, Germany), and cloned into the pBK3 fluorescence protein expression vector to screen for error-free gene constructs^37^. *E. coli* clones containing error-free gene constructs were selected by fluorescence, and sequences were verified by Sanger sequencing (MacroGen, Korea).

For the construction of plasmids containing engineered 1,4-BDO biosynthetic genes, we divided total pathway into two parts: the upstream pathway (*sucCD*, *sucD*, *4hbd*, and *kgd*) and the downstream pathway (*bld_M_* and *cat2*) according to 4HB synthesis. We amplified *sucCD* from the *E. coli* MG1655 genomic DNA and cloned it into the pTac15K vector, generating pTac15K sucCD. Synthesized *sucD* and *4hbd* genes, which were *E. coli* codon-optimized (Cosmogenetech, Korea) for efficient translation, were cloned into pTac15K sucCD using In-Fusion cloning (Clontech Laboratories, USA) to acquire pTac15K sucCD-sucD-4hbd. The vector including the entire upstream pathway, pTac15K sucCD-sucD-4hbd-kgd, was the produced by introducing the *kgd* gene prepared by PCR from *C. glutamicum* (ATCC 13032) genomic DNA into pTac15K sucCD-sucD-4hbd by In-Fusion cloning. To construct the downstream pathway plasmid, *E. coli* codon-optimized *cat2* gene (Cosmogenetech) was cloned into pTrc99A (Amersham Pharmacia Biotech, USA), producing pTrc99A cat2. Following the *cat2* cloning, the mutated *bld* gene which constains M227L and L273I mutation was cloned to pTrc99A cat2 to generate pTrc99a bld_M_-cat2. The *bld* gene was amplified from genomic DNA of *C. saccharoperbutylacetonicum*, and M277L and L273I mutations were introduced by site-directed mutagenesis^24^. Sequences of oligos for construction of engineered 1,4-BDO biosynthetic pathway plasmids are listed in Supplementary Table 10.

### Electroporation and λ Red recombination

All oligos and dsDNA substrates for genomic integration were electroporated into *E. coli* using the standard recombineering protocols^38^. Briefly, *E. coli* cells were grown at 30°C until mid-log phase (O.D. 600 nm = ~0.6). Cultures were then incubated at 42°C for 15 min to induce expression of the λ Red proteins, and cells were harvested and washed with ice-cold distilled water twice to remove salts. Washed cells were then resuspended in 50 μl of ice-cold water with either 10 μM DNA oligos or 1,000 ng of dsDNA substrates. Reactions were electroporated with a pulse of 1.8 kV in 1-mm gap cuvettes, and cells were recovered in 3ml of fresh Luria Bertani (LB) media at 30°C. Electroporations were conducted once or twice to increase the probability of recombination.

### Positive selection with *tolC*

For positive selection, cells were recovered for 3 hr after the final round of electroporation. These were then grown at 30°C for approximately 16 hr on LB plates solidified with 1.5% agar (Becton, Dickinson and Company, USA) containing 10 g/L tryptone (Becton, Dickinson and Company, USA), 5 g/L yeast extract (Becton, Dickinson and Company, USA), and 10 g/L NaCl (Duksan, Korea). SDS, 0.01% (w/v), (Fluka, USA) and 100 ng/μl spectinomycin (Duchefa Biochemie, Netherlands), a resistance marker for pN249 needed for transcription of *T7* promoter for positive selection, were added to the LB agar plates for positive selection.

### Negative selection with *tolC*

For negative selection, cells were incubated for 16 hr after the final round of electroporation to prevent *tolC* transcription before selection. These were then grown at 30°C for 16 hr on LB agar plates supplemented with 100 ng/μl spectinomycin (Duchefa Biochemie) and 0.01% (v/v) Colicin E1 extracted from JC411 (ATCC 27138). Colicin E1 was prepared as reported previously^39^.

### Verification of target insertion

Colony PCR was performed to confirm correct insertion of DNA fragments; 20 μl reactions were prepared with 10 μl 2x *taq* PCR premix (Intron, Korea), 1 μl each of 10 μM forward and reverse primers, and 1 μl template. Colony PCR conditions were as follows: Step 1 - 3 min at 95°C; Step 2 - 30 sec at 95°C; Step 3 - 30 sec at 60°C; Step 4 - 30 sec per 500 bp at 72°C; Step 5 - repeat steps 2 - 4 34 times; Step 6 - 10 min at 72°C. Sequences of primers for colony PCR are listed in Supplementary Table 9.

### Sequencing verification

Following the insertion of each DNA substrate, colony PCR was performed to amplify the integrated section. The size of the inserted fragment was verified with agarose gel electrophoresis, the product band was purified using the QIAquick Gel Extractionkit (Qiagen), and the sequence was verified by Sanger sequencing (MacroGen). After genomic insertion of the whole 1,4-BDO biosynthetic pathway, the genome sequence was verified by Illumina sequencing. Briefly, genomic DNA was prepared using a Genomic DNA Extraction Kit (Qiagen), followed by shearing to 300 bp (Covaris M220). After shearing, DNA was purified and concentrated to 40 μl final volume using a QIAquick PCR purification kit (Qiagen). Gel electrophoresis was performed to check the size of the bands, and only those in the range of 300–600 bp were excised and purified using QIAquick Gel Extraction kit (Qiagen). Samples for Illumina sequencing were prepared by NEBNext modules (NEBNext End Repair Module®, NEBNext dA-Tailing Module®, NEBNext Quick Ligation Module®, NEBNext Multiplex Oligo for Illumina®, New England Biolab, USA). The purified sample was then sent for Illumina sequencing (MacroGen), and the data were mapped to reference sequence by CLC Genomics Workbench (version 6.5.1).

### Production and measurement of 1,4-butanediol from glucose

In order to measure production of 4HB (an intermediate of the 1,4-BDO pathway), GBL (a lactonized form of 4HB), and 1,4-BDO, we pre-cultured a single bacterial colony in LB media with appropriate antibiotics overnight. This was inoculated at a concentration of 1% in 30 ml of a synthetic-defined MR media^40^, which is supplemented with 20 g/L glucose as carbon source, 0.8 g MgSO_4_·7H_2_O, 1 g yeast extract, 100 mM MOPS, and 10 mM NaHCO_3_ per liter. This culture was incubated anaerobically for 48 hr at 30°C and 220 rpm. Anaerobic cultivation was performed using 125 mL Erlenmeyer flasks with screw caps. An Acquity UPLC (Waters corp., USA) was coupled with Quattro Premier XE tandem mass spectrometery to measure the production level of 1,4-BDO, 4HB, and GBL. Each was quantified using a HSS T3 column (1.8 μm, 2.1 × 100 mm). Each sample was harvested after 48 hr of fermentation, and 5 μl of supernatant diluted was injected and separation was allowed to proceed at a fixed flow rate of 0.5 ml/min, at 65°C. Gradient mode (A: 0.2% formic acid in water/B: methanol) of mobile phase was applied for positive ionization^41^ and multiple reaction monitoring (MRM) was performed.

## Author Contributions

J.J., H.N.S., Y.K.J., J.H.P. and D.B. conceived the project. J.J., H.N.S. and Y.K.J. performed all the experiments and data analysis. J.L., G.R., W.L., E.K., K.R. and J.K. contributed to construction of strains. H.-Y.C. conducted analysis. J.H.P. and D.B. jointly supervised the research. J.J., H.N.S., Y.K.J., K.M.C., J.H.P. and D.B. wrote the paper.

## Supplementary Material

Supplementary Information Supplementary Info

## Figures and Tables

**Table 1:** Strains and plasmids used in this study

Designation	Genotype or description	References
*E. coli* Strains		
K-12 MG1655	*E. coli* K-12 MG1655 wild-type	
EcNR2	As K-12 MG1655 plus *bioA/bioB::*λ-prophage, *mutS::ampR*	Wang *et* *al*, *Nature*, 2009
YSB11	As EcNR2 plus *ΔldhA, ΔpflB, ΔadhE, lpdA::K.lpd* E354K, *Δmdh, ΔarcA, gltA* R164L	This study
YSB20	As YSB11 plus *P_p1_ald*, *P_p2_4HBd*, *P_p3_cat2*, *P_p4_sucD* at *P_tolC_*	This study
YSB25	As YSB11 plus *P_tac_*, *sucCD, sucD, 4HBd, kgd at P_tolC_*	This study
YSB27	As YSB25 plus *P_trc_*, *bld_M_* (*bld* M227L, L273I), *cat2 at P_tolC_*	This study
W	*E. coli* W wild-type	Archer *et al*, *BMC Genomics*, 2011
W023	As W plus *ΔldhA, ΔpflB, ΔadhE, lpdA::K.lpd* E354K, *Δmdh, ΔarcA, gltA* R164L	Hwang *et* *al*, *Biotechnology and Bioengineering*, 2014
W029	As W023 plus *ΔgabD, Δsad, P_acs_*::*P_trc_*:*acs, ΔpuuC, P_ppc_::P_trc_:ppc*	This study
W029-7	As W029 plus *P_tac_*, *sucCD, sucD, 4HBd, kgd at P_tolC_*	This study
W029-11	As W029-7 plus *P_trc_*, *bld_M_, cat2 at P_tolC_*	This study
W029-12	As W029-7 plus pTrc99a bld_M_-cat2	This study
W029-13	As W029 plus pTac15k sucCD-sucD-4hbd-kgd, pTrc99a bld_M_-cat2	This study
Plasmids		
pTac15k	*Km^R^*, *tac* promoter, p15A origin	Qian *et* *al*, *Biotechnology and Bioengineering*, 2009^42^
pTrc99a	*Ap^R^*, *trc* promoter, pColE1 origin	Amersham Pharmacia
pTac15k sucCD-sucD-4hbd-kgd	As pTac15k plus *sucCD* *from E. coli*, *sucD* and *4hbd* from *P. gingivalis*, *kgd* from *C. glutamicum*	This study
pTrc99a bld_M_-cat2	As pTrc99a plus *cat2* from *P. gingivalis* and *bld_M_* (*bld* M227L, L273I) from *C. saccharoperbutylacetonicum*	This study
pEKEx2	*Km^R^*, pBL1 origin	Eikmanns *et* *al*. *Gene*, 1991^43^
pEKEx2-Red	As pEKEx2 plus λ-prophage	This study

**Table 2:** Production of 4HB, GBL, and 1,4-BDO by engineered strains

E. coli strains	4HB (mg/L)	1,4-BDO (mg/L)	GBL (mg/L)
W029-7	773.33 ± 196.52	N/A	N/A
W029-11	1020.00 ± 109.56	33.33 ± 1.83	426.67 ± 30.71
W029-12	400.00 ± 53.59	1076.67 ± 91.63	33.33 ± 4.12
W029-13	370.00 ± 75.50	2790.20 ± 226.79	297.33 ± 15.55

Following 48 hr of anaerobic incubation, 4HB, GBL, and 1,4-BDO production levels were measured. GBL is produced spontaneously from 4HB-CoA or by the conversion of 4HB in acidic conditions. W029-7 does not contain the downstream pathway needed to generate 4HB-CoA, and all fermentation conditions were performed at neutral pH; therefore, it could not produce GBL. Each test was performed in triplicate, and the results are presented as mean ± s.d.
