# Supplementary material for: Repetitive genomic insertion of gene-sized dsDNAs by targeting the promoter region of a counter-selectable marker
Source: Sci Rep. 2015 Mar 4;5:8712. doi: 10.1038/srep08712 (PMC4348660; doi:10.1038/srep08712)
Supplement: Supplementary Information — Supplementary Info [file srep08712-s1.pdf]

Supplementary Information for:

Repetitive genomic insertion of gene-sized dsDNAs by targeting the promoter region of a counter-selectable marker

Jaehwan Jeong[1,§], Han Na Seo[1,§], Yu Kyung Jung[2,§], Jeewon Lee[1], Gyuri Ryu[1], Wookjae Lee[1], Euijin Kwon[1], Keunsoo Ryoo[1], Jungyeon Kim[3], Hwa-Young Cho[2], Kwang Myung Cho[2], Jin Hwan Park[2,*], Duhee Bang[1,*]

1. Department of Chemistry, Yonsei University, 50 Yonsei-ro, Seodaemun-gu, Seoul 120-749, Korea,
2. Samsung Advanced Institute of Technology, 130 Samsung-ro, Yeongtong-gu, Suwon-si, Gyeonggi-do, 443-803, Korea
3. Department of Chemistry, University College London, 20 Gordon Street, London, WC1H 0AJ, U.K.

[§]These authors contributed equally to this work.

*Corresponding authors.
E-mail address : duheebang@yonsei.ac.kr, jh111.park@samsung.com

Supplementary Note 1. Genomic insertion of engineered 1,4-BDO biosynthetic pathway in YSB11.

Insertion of the 1,4-BDO biosynthetic pathway into the genome of YSB11 was accomplished by dividing the upstream pathway genes into 2,340 bp, 2,048 bp, 2,280 bp, and 2,312 bp fragments. Through λ red recombination, the 2,340 bp substrate was first incorporated into the *tolC* promoter region, resulting in the deletion of this promoter. Utilizing negative selection, we identified a clone with the 2,340 bp fragment correctly inserted. We then introduced the 2,048 bp substrate, which restored the *tolC* promoter and was isolated using positive selection. Insertion of the next 2,280 bp substrate disrupted the *tolC* promoter region and was isolated with negative selection. The fourth insertion of the 2,312 bp substrate, which again was designed to restore the *tolC* promoter, was repeated several times using λ red recombination. However, due to the reduced efficiency of *tolC* selection, we were unable to isolate clones containing the correct insertion. We therefore divided the 2,312 bp substrate into two parts, resulting in fragments of 652 bp and 1,600 bp. The 652 bp insertion was obtained using positive selection, and subsequently, through negative selection, we were able to obtain the final insertion of the 1,600 bp fragment and create strain YSB25, which encodes the entire 1,4-BDO upstream pathway.

To incorporate the downstream pathway, we prepared two substrates of 1,482 bp and 1,742 bp. The 1,482 bp and 1,742 bp fragments were inserted into the *tolC* promoter of strain YSB25 through positive selection and negative selection, respectively. In this manner, we achieved insertion of the entire 1,4-BDO biosynthetic pathway into the K-12 MG 1655-derived *E. coli* strain YSB27 (Supplementary Fig. 3).

Supplementary Note 2. Development of the W029 strain.

Deletion of the *gabD*, *sad*, and *puuC* genes from the chromosome was performed using a one-step inactivation method [1]. Substitution of the native promoters of *acs* and *ppc* genes by the *trc* promoter was performed by PCR-mediated λ Red recombination [2].

For the deletion of the *gabD* gene, a PCR substrate containing the lox71 site, the chloramphenicol resistance gene (*cat*), and the lox66 site was prepared. This dsDNA substrate (1,234 bp) was generated by PCR with primers gabDF and gabDR using plasmid pMloxC [3] as a template (sequences of oligonucleotides and primers are listed in Supplementary Table 11). The gel purified PCR product was electroporated into *Escherichia coli* harbouring the pKD46 plasmid, which expresses the λ Red proteins [1]. The mutants in which gene inactivation occurred by double homologous recombination were selected on LB agar containing 20 μg/mL chloramphenicol and were subsequently screened by direct colony PCR. Deletion of the *sad* and *puuC* genes was performed with the following primers in the same manner as described for the *gabD* gene deletion: sadF and sadR for the *sad* gene; puuCF and puuCR for the *puuC* gene.

To construct marker-free mutant strains, the antibiotic selection marker was eliminated by using a helper plasmid, pJW168, containing the Cre recombinase, which recognizes lox sequences, and an ampicillin resistance gene. The chloramphenicol resistant mutants were transformed with the pJW168 [4], and ampicillin-resistant transformants were selected at 30°C on LB agar plates containing 100 μg/mL ampicillin and 1 mM isopropyl β-*D*-1-thiogalactopyranoside (IPTG, Sigma-Aldrich, USA) to induce expression of Cre recombinase . Colonies that lost chloramphenicol resistance were screened by colony PCR. Positive colonies were cultivated in LB medium without antibiotic markers at 42°C to cure the temperature-sensitive plasmids, and these were then were examined for the loss of all antibiotic resistance markers.

The PCR substrate for replacement of the native *acs* promoter with the *trc* promoter by homologous recombination was prepared by PCR with primers RPacsF and RPacsR, using plasmid pMtrc9 as a template. This produced a dsDNA fragment (1,624 bp) containing the *trc* promoter downstream of lox71-*cat*-lox66 cassette, which was electroporated into *E. coli* expressing the λ Red proteins [1]. The mutants in which promoter replacement occurred by double homologous recombination were selected on LB agar containing 20 μg/mL chloramphenicol and were subsequently screened by direct colony PCR. Substitution of the native promoter of *ppc* gene by the *trc* promoter was performed with the primers RPppcF and RPppcR in the same manner as described for the *acs* gene. The antibiotic selection marker was eliminated by using the helper plasmid, pJW168 [4], as described above.

Supplementary Note 3. Counter selection based on *thyA*.

Thymidylate synthase A (*thyA*) encodes an enzyme essential for the synthesis of thymidine monophosphate (dTMP) from deoxyuridine monophosphate (dUMP), which is subsequently phosphorylated to thymidine triphosphate (dTTP). Cells with *thyA* knocked out are unable to synthesize DNA and undergo thymine-less death, thus enabling positive selection of cells incorporating *thyA* on minimal M9 broth, which does not include thymine. Thymidylate synthase A requires tetrahydrofolate (THF), along with dUMP, for the methylation of dUMP to form dTMP, yielding dihydrofolate (DHF) as a residual product. THF is essential for other cellular reactions as well and needs to be replenished from DHF by dihydrofolate reductase, which is inhibited by trimethoprim. Thus, cells with dihydrofolate reductase repressed are depleted of THF. That is, cells without the *thyA* gene can be selected in minimal M9 broth supplemented with thymine and trimethoprim. Therefore, in order to perform *thyA* counter-selection, positive selection was conducted in the absence of thymine, and negative selection was conducted in the presence of thymine and trimethoprim.

For counter selection of *thyA*, cells were grown in minimal M9 broth containing 42 mM Na_2HPO_4, 24 mM KH_2PO_4, 9 mM NaCl, 19 mM NH_2Cl, 1 mM $MgSO_4$, 0.1 mM $CaCl_2$, 2% glucose, and 0.5 µg/ml thiamine. Minimal broth was supplemented with 0.6 mM leucine, valine, and isoleucine, as well as 1% (v/v) LB broth and 2.5% (w/v) agar (Becton, Dickinson and Company); 100 ng/µl Spectinomycin (Buchefa Biochemie) was also added for pN249 maintenance. For positive selection of *thyA,* M9 minimal salts medium was supplemented with 0.79 mM thymine, for negative selection 20 µg/mL trimethoprim was added [5].

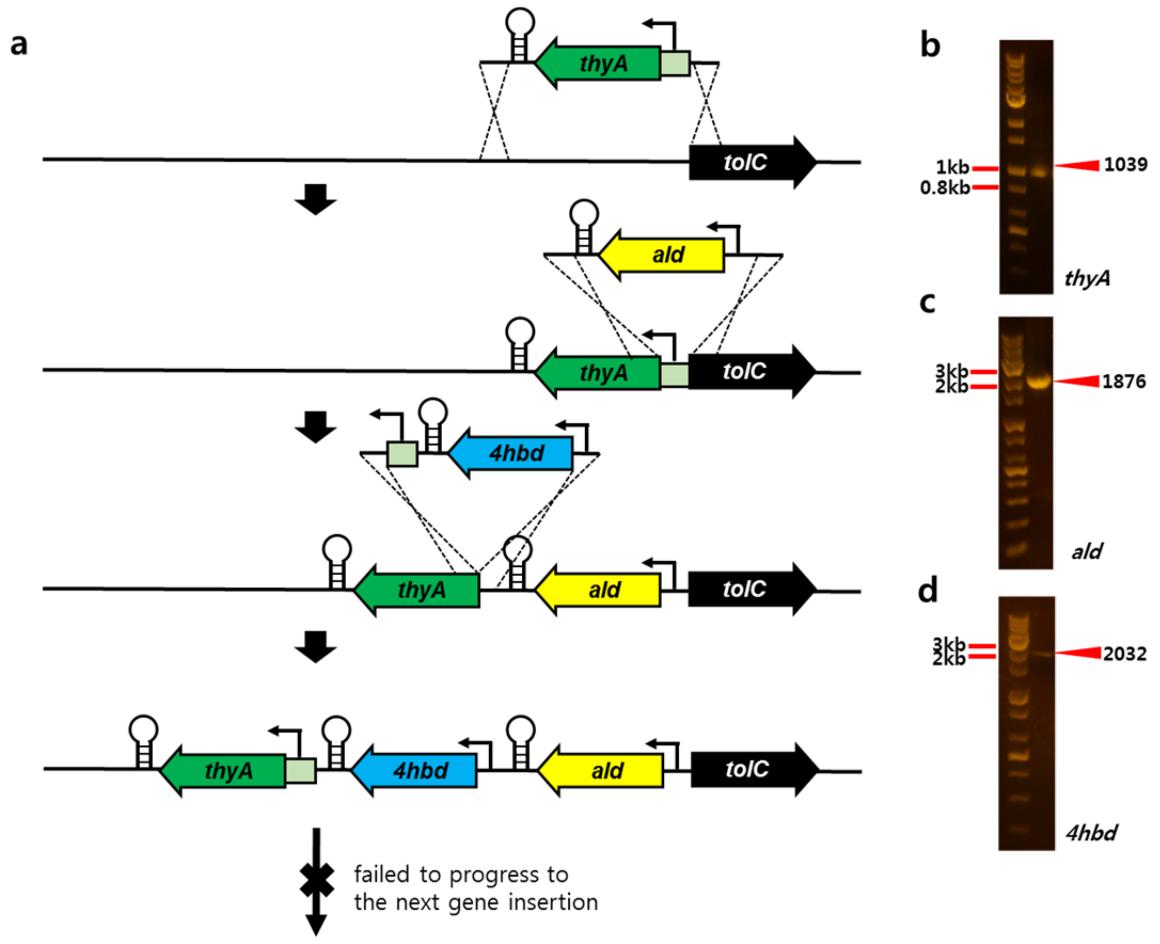

Supplementary Figure 1. Insertion of 1,4-BDO pathway genes using *thyA* counter selection. (**a**) A schematic representation of the insertion of the 1,4-BDO gene cluster using a counter-selectable *thyA* gene marker. The *thyA* gene, regulated by a *T7* promoter, was incorporated next to the *tolC* gene, since the original *thyA* gene is next to the essential gene, *lgt*, where the insertion of the BDO gene cluster was planned to take place. Using *thyA* selection, the original *thyA* was knocked out, and *thyA*, *ald,* and *4HBd* were all successfully and efficiently incorporated. However, after two rounds of counter selection, the efficiency of selection dropped to zero. Panel (**b**) depicts insertion of *thyA*, panel (**c**) depicts insertion of *ald*, and panel (**d**) depicts insertion of *4hbd*.

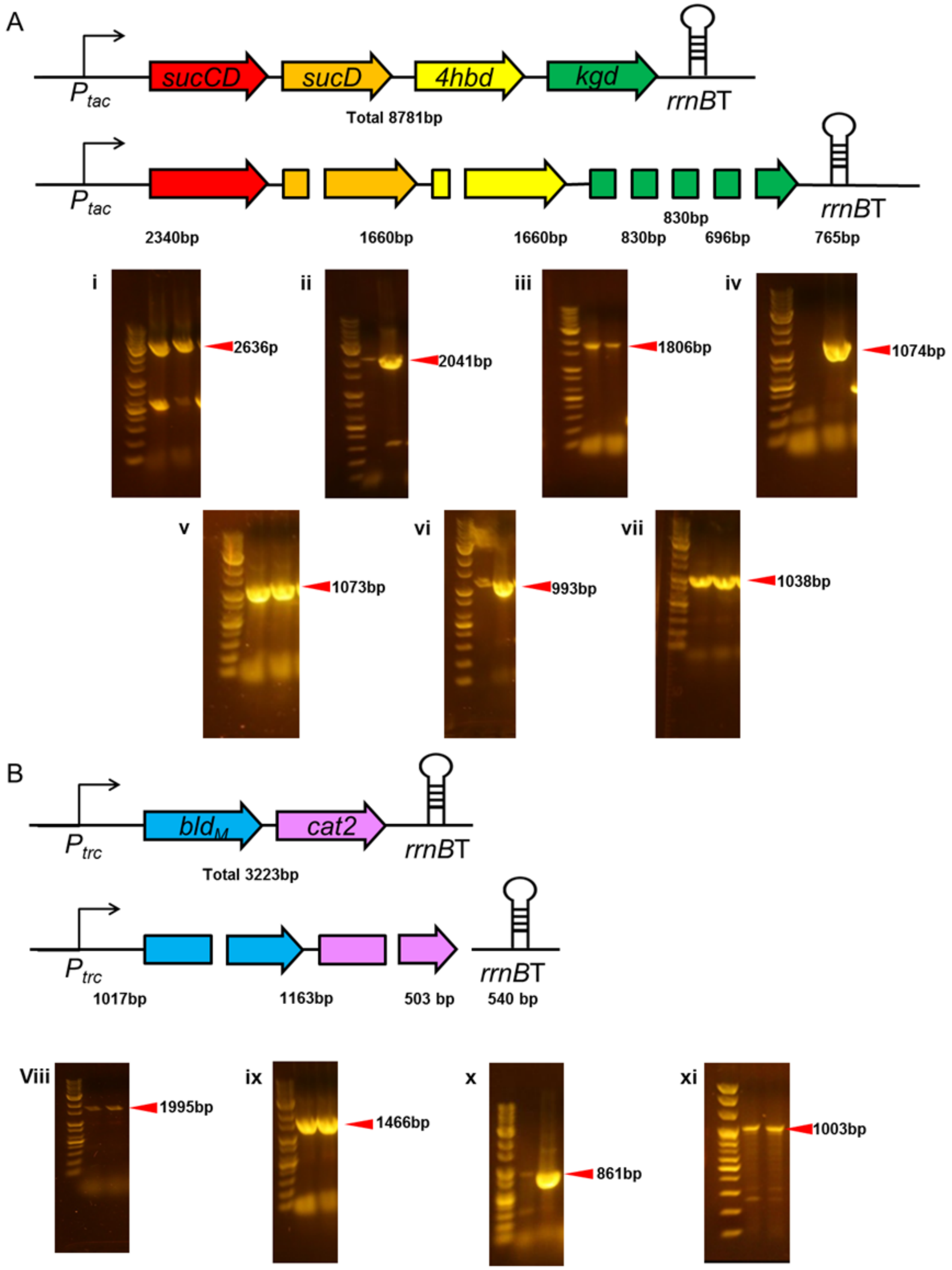

Supplementary Figure 2. Genomic insertion of the engineered 1,4-BDO pathway genes into W029. A schematic representation of the insertion of an engineered 1,4-BDO biosynthetic pathway in *E. coli* strain W029. Panel (**a**) represents the upstream pathway and

the seven divided substrates utilized to construct W029-7. Sub-panels (i) through (vii) show colony PCR results for each upstream fragment insertion. Panel (**b**) represents the downstream pathway and the two substrates utilized to construct W029-11. Sub-panels (viii) through (xi) show colony PCR results for each downstream fragment insertion.

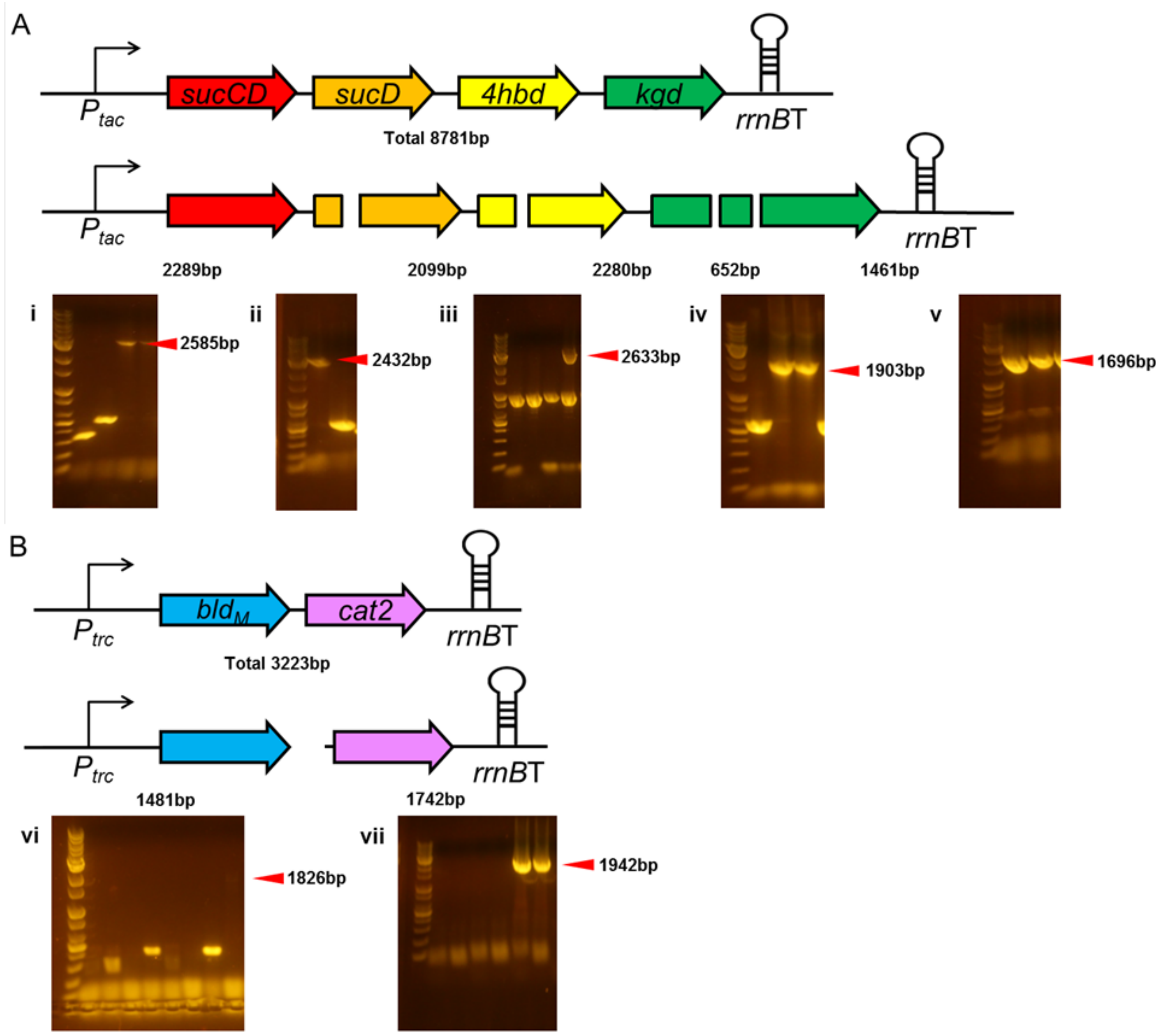

Supplementary Figure 3. Genomic insertion of the engineered 1,4-BDO pathway genes into YSB11. Panel (**a**) represents the upstream pathway and the five divided substrates utilized to construct YSB25. Sub-panels (i) through (v) show colony PCR results for each upstream fragment insertion. Panel (**b**) represents the downstream pathway and the two divided substrates utilized to construct YSB27. Sub-panels (vi) through (vii) show colony PCR results for downstream fragment insertion.

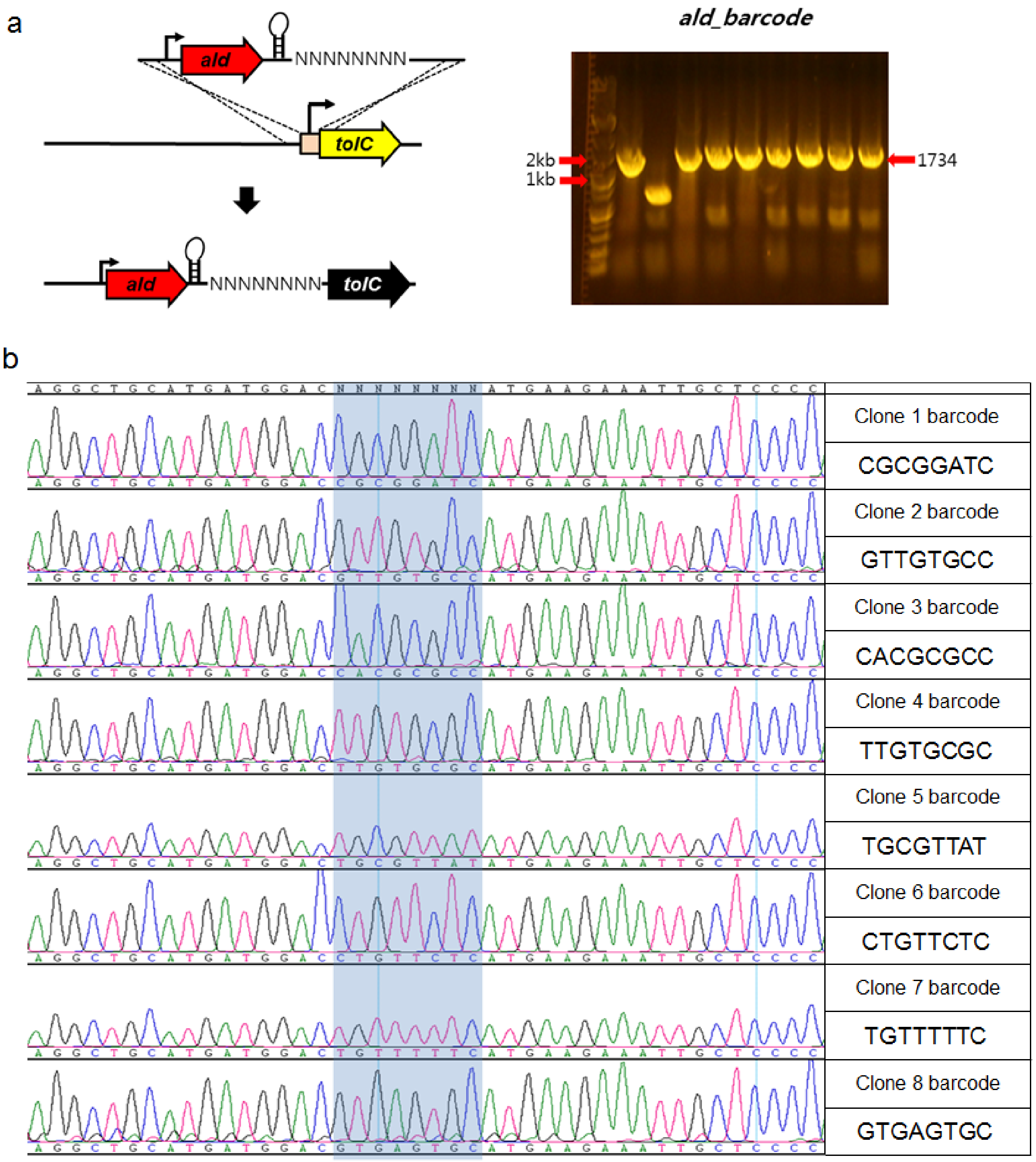

Supplementary Figure 4. Genomic insertion of *ald* with unique barcode sequences. (**a**) Insertion of *ald* with 8 bp unique barcode sequences was performed through negative selection. (**b**) Barcode sequences of 8 randomly selected *E.coli* clone showed all different sequences. Barcode regions are highlighted.

Supplementary Table 1. Oligonucleotides for knock-out to construct YSB11. Each oligonucleotide was designed to contain the desired mutation in the middle of the oligo, which was 90 nucleotides of length. Mutations are highlighted in orange. After the introduction of knock-out oligos, the target genes contained a TAAT truncation into 100 bp from the start of the open reading frame.

Name	Sequences (5` → 3`)
pflB KO	CCTGTGGGACAAAGTAATGGAAGGCGTTAAACTGGAAAACCGCTAATACT CACGCGCCAGTTGACTTTGACACCGCTGTTGCTTCCACCA
mdh KO	TACGCGCTACGCCTGCAGAGATAAGAACGACATCTGCGCCTTCATTACAG CGCCGGAGTCGCATCTTCACCAGAAAAACCTTTGATTTTC
ldhA KO	CGATGACGGCAGCCGCCCGGTGCTGGAAGAGCTGAAAAAGCACTAATGG CGTTAAATATATCGCCCTGCGCTGTGCCGGTTTCAATAACG
arcA KO	GGATATCAATCTGCCGGGTAAGAACGGTCTTCTGTTAGCGCGTTAATGAA CTGCGCGAGCAGGCGAATGTTGCGTTGATGTTCCTGACTG
adhE KO	CGTTGCCGAATCCGGCATGGGTATCGTCGAAGATAAAGTGATCTAATAAA AACCACTTTGCTTCTGAATATATCTACAACGCCTATAAAG
gltA R164L	CGCTGGATGTTAACAATCCTCGTCACCGTGAAATTGCCGCGTTCTTACTG CTGTCGAAAATGCCGACCATGGCCGCGATGTGTTACAAGT

Supplementary Table 2. DNA sequences of 1,4-BDO biosynthetic pathway genes. Sequences of *sucD*, *4hbd* and *cat2* are from *P. gingivalis*, *ald* is from *C. beijerinckii*, and *sucA* is from *M. bovis*. The sequences of *lpdA* are from *K. pneumoniae* and its sequence contains the E354K mutation.

Gene	Sequence (5` → 3`)
ald	ATGAATAAAGACACACTGATCCCTACAACTAAAGATTTAAAAGTAAAAAC<br>AAATGGTGAAAACATTAATTTAAAGAACTACAAAGATAATAGCAGTTGTTT<br>CGGCGTATTCGAAAATGTTGAAAATGCTATCAGCAGCGCTGTACACGCA<br>CAAAAGATATTATCGCTGCATTATACAAAAGAGCAACGTGAAAAAATCAT<br>CACTGAGATACGTAAGGCCGCATTACAAAATAAAGAGGTGCTGGCTACA<br>ATGATTCTGGAAGAAACACATATGGGACGTTATGAGGATAAAATATTAAA<br>ACATGAACTGGTAGCTAAATATACTCCTGGTACAGAAGATTTAACTACTA<br>CTGCCTGGAGCGGTGATAATGGTCTGACAGTTGTAGAAATGTCTCCATA<br>TGGTGTTATTGGTGCAATAACTCCTTCTACCAATCCAACTGAAACTGTAA<br>TTTGTAATAGCATTGGCATGATTGCTGCTGGAAATGCTGTAGTATTTAAC<br>GGACACCCATGCGCTAAAAAATGTGTTGCCTTTGCTGTTGAAATGATCAA<br>TAAGGCAATTATTAGCTGTGGCGGTCCGGAAAATCTGGTAACAACTATAA<br>AAAATCCAACCATGGAGTCTCTGGATGCCATTATTAAGCATCCTTCAATA<br>AAACTGCTTTGCGGAACTGGCGGTCCAGGAATGGTAAAAACCCTGTTAA<br>ATTCTGGTAAGAAAGCTATTGGTGCTGGTGCTGGAAATCCACCAGTTATT<br>GTCGATGATACTGCTGATATTGAAAAGGCTGGTCGTAGCATCATTGAAG<br>GCTGTTCTTTTGATAATAATTTACCTTGTATTGCAGAAAAAGAAGTATTTG<br>TTTTTGAGAATGTTGCAGATGATTTAATATCTAACATGCTGAAAAATAATG<br>CTGTAATTATCAATGAAGATCAGGTATCAAAATTAATCGATTTAGTATTAC<br>AAAAAAATAATGAAACTCAAGAATACTTTATCAACAAAAAATGGGTAGGT<br>AAAGATGCAAAATTATTCCTCGATGAAATCGATGTTGAGTCTCCTTCAAA<br>TGTTAAATGCATTATCTGCGAAGTGAATGCCAATCATCCATTTGTTATGA<br>CAGAACTGATGATGCCAATATTGCCAATTGTGCGCGTTAAAGATATCGAT<br>GAAGCTATTAAATATGCAAAGATTGCAGAACAAAATAGAAAACATAGTGC<br>CTATATTTATAGCAAAAATATCGACAACCTGAATCGCTTTGAACGTGAAA<br>TCGATACTACTATTTTTGTAAAGAATGCTAAATCTTTTGCTGGTGTTGGTT

	ATGAAGCAGAAGGATTTACCACTTTCACTATTGCTGGATCTACTGGTGAG GGCATAACCTCTGCACGTAATTTTACCCGCCAACGTCGCTGTGTACTGG CCGGCTAA
4hbd	ATGCAACTTTTCAAACTCAAGAGTGTAACACATCACTTTGACACTTTTGC AGAATTTGCCAAGGAATTCTGTCTTGGAGAACGCGACTTGGTAATTACCA ACGAGTTCATCTATGAACCGTATATGAAGGCATGCCAGCTCCCCTGCCA TTTTGTTATGCAGGAGAAATATGGGCAAGGCGAGCCTTCTGACGAAATG ATGAATAACATCTTGGCAGACATCCGTAATATCCAGTTCGACCGCGTAAT CGGTATCGGAGGAGGTACGGTTATTGACATCTCTAAACTTTTCGTTCTGA AAGGATTAAATGATGTACTCGATGCATTCGACCGCAAAATACCTCTTATC AAAGAGAAAGAACTGATCATTGTGCCCACAACATGCGGAACGGGTAGCG AGGTGACGAACATTTCTATCGCAGAAATCAAAAGCCGTCACACCAAAAT GGGATTGGCTGACGATGCCATTGTTGCAGACCATGCCATCATCATACCT GAACTTCTGAAGAGCTTGCCTTTCCACTTCTACGCATGCAGTGCAATCG ATGCTCTTATCCATGCCATCGAGTCATACGTATCTCCTAAAGCCAGTCCA TATTCTCGTCTGTTCAGTGAGGCGGCTTGGGACATTATCCTGGAAGTATT CAAGAAAATCGCCGAACACGGCCCTGAATACCGCTTCGAAAAGCTGGG AGAAATGATCATGGCCAGCAACTATGCCGGTATAGCCTTCGGAAATGCA GGAGTAGGAGCCGTCCACGCACTATCCTACCCGTTGGGAGGCAACTAT CACGTGCCGCATGGAGAAGCAAACTATCAGTTCTTCACAGAGGTATTCA AAGTATACCAAAAGAAGAATCCTTTCGGCTATATAGTCGAACTCAACTGG AAGCTCTCCAAGATACTGAACTGCCAGCCCGAATACGTATATCCGAAGC TGGATGAACTTCTCGGATGCCTTCTTACCAAGAAACCTTTGCACGAATAC GGCATGAAGGACGAAGAGGTAAGAGGCTTTGCGGAATCAGTGCTTAAG ACACAGCAAAGATTGCTCGCCAACAACTACGTAGAGCTTACTGTAGATG AGATCGAAGGTATCTACAGAAGACTCTACTAA
cat2	ATGCAATGGCAAGAACTTTACCGTCAGCGCGTTTGCTCTGCAGACGAAG CTGTCGTGGACTCTCTTAAACCGGGAACGAAAGTTGTATTCGGTCATGC TGCTGCTGCGCCTGTCCGTTTCTCTCAGGCTATGTACCGCCAGCGTGAA AAGTTGGAGAATATCACAGTTTTCCACATGTTGTATTTCGGCGACGCGC CGCACCTTGCTCCCGAAATGCGTTCGCATGTACACCCGACTCTCAACTT

	CCTTGAGGGCAACTCCCGTCCGGCAAGCCGTGACCGTCGTGTCGATTT CATTCCCTGCCACTTCCACGAGGTACCGGAACTGTTTCGTCAGGGATTC TTTCCATTGGATGTAGCCGTAGTGCAGGTATCTACTCCTAACGAAGAGG GTTATTGCTCTTTCGGAGTTTCCTGCGACTACACAAAGGCTGCCGCCGA GTGCGCTCCGGTAGTAGTAGCCGAGGTGAACAAGCAAATGCCATTCATC GGTGGTGAAAACCTGATTCACATCTCCAAACTGACCCATATCATCGAAGT GGACGAGCCGATTGCAGAAGTATTGCCTCCTGCTATCAGCGACCTTGAA CTGAGGATAGGTCAGAATTGTGCCTCACTGATCAAAGACGGCGATACCC TCCAGTTGGGTATCGGCGGTATCCCCGACGCTGTGTTGCGTGCATTGG AAGGGCATAAAGATCTCGGTATTCACACGGAAATGTTTACCGACGGGGT GATGCGTATGATTCGCAAGGGGATTATCAACGGGAAGAAAAAAACATTG CATCCCGAAAAAGTCGTTACCTCGCTAATCTTCGGATCGAAAGAATTGTA CGATTTTGTCAATAACAATCCGGTGATAGAATGCTATCCGGTGGATTATA TCAACAACCCCGATGTTATCGGTAAGAATGACCGCATGGTTTCTATCAAT TCCTGCTTGGAGATGGATCTCATGGGGCAGGCAGCTTCTGAGTCGATC GGGTACGAACAGTTCAGTGGATCCGGAGGTCAAGTCGATTTCCTTCGTG GGGCCAAGCGTTCCAAGGGAGGAATCTCCATTATGGCTTTCCCCAGTAC GGCCAAGAAAGGGACTGAGAGTCGCATCGTTCCCATTCTGAAAGAGGG TGCTTGTGTCACGACCGGCCGTAACGAAGTGGACTATGTGGTGACGGA ATATGGCGTAGCGCGTCTGCGTGGCGCAACGCTTCGTCAGCGTGCTGA AGCCTTGACTGCTATAGCACATCCCGATTTCCGACCGGCCCTTGAGGAG GAAATCCGCCGACGCTTCGAATAA
sucD	ATGGAAATCAAAGAAATGGTGAGCCTTGCACGCAAGGCTCAGAAGGAGT ATCAAGCTACCCATAACCAAGAAGCAGTTGACAACATTTGCCGAGCTGC AGCAAAAGTTATTTATGAAAATGCAGCTATTCTGGCTCGCGAAGCAGTAG ACGAAACCGGCATGGGCGTTTACGAACACAAAGTGGCCAAGAATCAAG GCAAATCCAAAGGTGTTTGGTACAACCTCCACAATAAAAAATCGATTGGT ATCCTCAATATAGACGAGCGTACCGGTATGATCGAGATTGCAAAGCCTA TCGGAGTTGTAGGAGCCGTAACGCCGACGACCAACCCGATCGTTACTC CGATGAGCAATATCATCTTTGCTCTTAAGACCTGCAATGCCATCATTATT GCCCCCCACCCCAGATCCAAAAAATGCTCTGCACACGCAGTTCGTCTGA

	TCAAAGAAGCTATCGCTCCGTTCAACGTACCGGAAGGTATGGTTCAGAT CATCGAAGAACCCAGCATCGAGAAGACGCAGGAACTCATGGGCGCCGT AGACGTAGTAGTTGCTACGGGTGGTATGGGCATGGTGAAGTCTGCATAT TCTTCAGGAAAGCCTTCTTTCGGTGTTGGAGCCGGTAACGTTCAGGTGA TCGTGGATAGCAACATCGATTTCGAAGCTGCTGCAGAAAAAATCATCAC CGGTCGTGCTTTCGACAACGGTATCATCTGCTCAGGCGAACAGAGCATC ATCTACAACGAGGCTGACAAGGAAGCAGTTTTCACAGCATTCCGCAACC ACGGTGCATATTTCTGTGACGAAGCCGAAGGAGATCGGGCTCGTGCAG CTATCTTCGAAAATGGAGCCATCGCGAAAGATGTAGTAGGTCAGAGCGT TGCCTTCATTGCCAAGAAAGCAAACATCAATATCCCCGAGGGTACCCGT ATTCTCGTTGTTGAAGCTCGCGGCGTAGGAGCAGAAGACGTTATCTGTA AGGAAAAGATGTGTCCCGTAATGTGCGCCCTCAGCTACAAGCACTTCGA AGAAGGTGTAGAAATCGCACGTACGAACCTCGCCAACGAAGGTAACGG CCACACCTGTGCTATCCACTCCAACAATCAGGCACACATCATCCTCGCA GGATCAGAGCTGACGGTATCTCGTATCGTAGTGAATGCTCCGAGTGCCA CTACAGCAGGCGGTCACATCCAAAACGGTCTTGCCGTAACCAATACGCT CGGATGCGGATCATGGGGTAATAACTCTATCTCCGAGAACTTCACTTAC AAGCACCTCCTCAACATTTCACGCATCGCACCGTTGAATTCAAGCATTCA CATCCCCGATGACAAAGAAATCTGGGAACTCTAA
lpdA	ATGAGTACTGAAATCAAAACTCAGGTCGTGGTACTTGGGGCAGGCCCCG CAGGTTACTCTGCAGCCTTCCGTTGCGCTGATTTAGGTCTGGAAACCGT CATCGTAGAACGTTACAGCACCCTCGGTGGTGTTTGTCTGAACGTGGGT TGTATCCCTTCTAAAGCGCTGCTGCACGTGGCAAAAGTTATCGAAGAAG CGAAAGCGCTGGCCGAACACGGCATCGTTTTCGGCGAACCGAAAACTG ACATTGACAAGATCCGCACCTGGAAAGAAAAAGTCATCACTCAGCTGAC CGGTGGTCTGGCTGGCATGGCCAAAGGTCGTAAAGTGAAGGTGGTTAA CGGTCTGGGTAAATTTACCGGCGCTAACACCCTGGAAGTGGAAGGCGA AAACGGCAAAACCGTGATCAACTTCGACAACGCCATCATCGCGGCGGG TTCCCGTCCGATTCAGCTGCCGTTTATCCCGCATGAAGATCCGCGCGTA TGGGACTCCACCGACGCGCTGGAACTGAAATCTGTACCGAAACGCATG CTGGTGATGGGCGGCGGTATCATCGGTCTGGAAATGGGTACCGTATAC

	CATGCGCTGGGTTCAGAGATTGACGTGGTGGAAATGTTCGACCAGGTTA<br>TCCCGGCTGCCGACAAAGACGTGGTGAAAGTCTTCACCAAACGCATCAG<br>CAAGAAATTTAACCTGATGCTGGAAACCAAAGTGACTGCCGTTGAAGCG<br>AAAGAAGACGGTATTTACGTTTCCATGGAAGGTAAAAAAGCACCGGCGG<br>AAGCGCAGCGTTACGACGCAGTGCTGGTCGCTATCGGCCGCGTACCGA<br>ATGGTAAAAACCTCGATGCAGGTAAAGCTGGCGTGGAAGTTGACGATCG<br>CGGCTTCATCCGCGTTGACAAACAAATGCGCACCAACGTGCCGCACATC<br>TTTGCTATCGGCGATATCGTCGGTCAGCCGATGCTGGCGCACAAAGGT<br>GTCCATGAAGGCCACGTTGCCGCAGAAGTTATCTCCGGTCTGAAACACT<br>ACTTCGATCCGAAAGTGATCCCATCCATCGCCTACACTGAACCAGAAGT<br>GGCATGGGTCGGTCTGACCGAGAAAGAAGCGAAAGAGAAAGGCATCAG<br>CTACGAAACCGCCACCTTCCCGTGGGCTGCTTCCGGCCGTGCTATCGC<br>TTCTGACTGCGCAGATGGTATGACCAAACTGATCTTCGACAAAGAGACC<br>CACCGTGTTATCGGCGGCGCGATTGTCGGCACCAACGGCGGCGAGCTG<br>CTGGGTGAGATCGGCCTGGCTATCGAGATGGGCTGTGACGCTGAAGAC<br>ATCGCCCTGACCATCCACGCTCACCCGACTCTGCACGAGTCCGTTGGC<br>CTGGCGGCGGAAGTGTTCGAAGGCAGCATCACCGACCTGCCAAACGCC<br>AAAGCGAAGAAAAAGTAA

Supplementary Table 3. DNA sequences of engineered 1,4-BDO biosynthetic pathway genes. The *sucCD* sequence is from *E. coli*. Sequences of *sucD*, *4hbd* and *cat2* are from *P. gingivalis.* Sequences of *kgd* and bld_M are from *C. glutamicum* and *C. saccharoperbutylacetonicum*, respectively. The *lpdA* sequence was also substituted by *lpdA* E354K from *K. pneumonia*, which is the same as the original design.

Gene	Sequence (5` → 3`)
sucCD	ATGAACTTACATGAATATCAGGCAAAACAACTTTTTGCCCGCTATGGC<br>TTACCAGCACCGGTGGGTTATGCCTGTACTACTCCGCGCGAAGCAGA<br>AGAAGCCGCTTCAAAAATCGGTGCCGGTCCGTGGGTAGTGAAATGTC<br>AGGTTCACGCTGGTGGCCGCGGTAAAGCGGGCGGTGTGAAAGTTGT<br>AAACAGCAAAGAAGACATCCGTGCTTTTGCAGAAAACTGGCTGGGCA<br>AGCGTCTGGTAACGTATCAAACAGATGCCAATGGCCAACCGGTTAAC<br>CAGATTCTGGTTGAAGCAGCGACCGATATCGCTAAAGAGCTGTATCT<br>CGGTGCCGTTGTTGACCGTAGTTCCCGTCGTGTGGTCTTTATGGCCT<br>CCACCGAAGGCGGCGTGGAAATCGAAAAAGTGGCGGAAGAAACTCC<br>GCACCTGATCCATAAAGTTGCGCTTGATCCGCTGACTGGCCCGATGC<br>CGTATCAGGGACGCGAGCTGGCGTTCAAACTGGGTCTGGAAGGTAA<br>ACTGGTTCAGCAGTTCACCAAAATCTTCATGGGCCTGGCGACCATTTT<br>CCTGGAGCGCGACCTGGCGTTGATCGAAATCAACCCGCTGGTCATCA<br>CCAAACAGGGCGATCTGATTTGCCTCGACGGCAAACTGGGCGCTGA<br>CGGCAACGCACTGTTCCGCCAGCCTGATCTGCGCGAAATGCGTGAC<br>CAGTCGCAGGAAGATCCGCGTGAAGCACAGGCTGCACAGTGGGAAC<br>TGAACTACGTTGCGCTGGACGGTAACATCGGTTGTATGGTTAACGGC<br>GCAGGTCTGGCGATGGGTACGATGGACATCGTTAAACTGCACGGCG<br>GCGAACCGGCTAACTTCCTTGACGTTGGCGGCGGCGCAACCAAAGA<br>ACGTGTAACCGAAGCGTTCAAAATCATCCTCTCTGACGACAAAGTGAA<br>AGCCGTTCTGGTTAACATCTTCGGCGGTATCGTTCGTTGCGACCTGA<br>TCGCTGACGGTATCATCGGCGCGGTAGCAGAAGTGGGTGTTAACGTA<br>CCGGTCGTGGTACGTCTGGAAGGTAACAACGCCGAACTCGGCGCGA<br>AGAAACTGGCTGACAGCGGCCTGAATATTATTGCAGCAAAAGGTCTG<br>ACGGATGCAGCTCAGCAGGTTGTTGCCGCAGTGGAGGGGAAATAAT

	GTCCATTTTAATCGATAAAAACACCAAGGTTATCTGCCAGGGCTTTAC CGGTAGCCAGGGGACTTTCCACTCAGAACAGGCCATTGCATACGGCA CTAAAATGGTTGGCGGCGTAACCCCAGGTAAAGGCGGCACCACCCA CCTCGGCCTGCCGGTGTTCAACACCGTGCGTGAAGCCGTTGCTGCC ACTGGCGCTACCGCTTCTGTTATCTACGTACCAGCACCGTTCTGCAAA GACTCCATTCTGGAAGCCATCGACGCAGGCATCAAACTGATTATCAC CATCACTGAAGGCATCCCGACGCTGGATATGCTGACCGTGAAAGTGA AGCTGGATGAAGCAGGCGTTCGTATGATCGGCCCGAACTGCCCAGG CGTTATCACTCCGGGTGAATGCAAAATCGGTATCCAGCCTGGTCACA TTCACAAACCGGGTAAAGTGGGTATCGTTTCCCGTTCCGGTACACTG ACCTATGAAGCGGTTAAACAGACCACGGATTACGGTTTCGGTCAGTC GACCTGTGTCGGTATCGGCGGTGACCCGATCCCGGGCTCTAACTTTA TCGACATTCTCGAAATGTTCGAAAAAGATCCGCAGACCGAAGCGATC GTGATGATCGGTGAGATCGGCGGTAGCGCTGAAGAAGCAGCTG CGTACATCAAAGAGCACGTTACCAAGCCAGTTGTGGGTTACATCGCT GGTGTGACTGCGCCGAAAGGCAAACGTATGGGCCACGCGGGTGCCA TCATTGCCGGTGGGAAAGGGACTGCGGATGAGAAATTCGCTGCTCTG GAAGCCGCAGGCGTGAAAACCGTTCGCAGCCTGGCGGATATCGGTG AAGCACTGAAAACTGTTCTGAAATAA
sucD	ATGGAAATAAAAGAGATGGTGTCGTTGGCAAGGAAAGCTCAGAAGGA ATATCAAGCGACCCATAATCAAGAAGCAGTTGATAACATTTGCCGAGC TGCAGCAAAAGTGATTTATGAAAATGCAGCTATACTGGCTCGCGAAG CAGTAGACGAAACCGGCATGGGCGTATATGAACATAAAGTGGCCAAG AATCAGGGGAAATCCAAAGGCGTCTGGTACAATTTGCACAATAAAAAA TCGATCGGTATCTTAAATATAGACGAGAGAACCGGGATGATCGAGAT AGCAAAACCTATCGGGGTTGTTGGAGCCGTAACCCCGACGACAAACC CGATTGTGACTCCAATGAGCAACATCATTTTTGCCCTTAAGACATGCA ATGCCATTATTATCGCCCCACATCCCAGATCCAAAAAATGCTCAGCAC ATGCAGTTCGTCTGATAAAGGAAGCAATCGCTCCGTTTAATGTCCCG GAGGGAATGGTTCAGATCATTGAAGAGCCCAGCATCGAGAAAACTCA GGAACTAATGGGCGCCGTGGATGTGGTAGTTGCGACGGGTGGTATG

	GGTATGGTGAAATCTGCATATTCTTCAGGGAAGCCTTCTTTTGGTGTA GGAGCCGGTAACGTTCAAGTGATCGTGGATAGTAATATCGATTTTGAA GCTGCGGCAGAAAAAATTATCACCGGCCGTGCTTTCGACAATGGGAT CATCTGTTCAGGCGAACAGAGTATCATCTACAACGAAGCTGACAAGG AAGCTGTCTTCACAGCCTTCCGCAACCATGGTGCATATTTTTGTGATG AAGCGGAGGGAGATCGGGCCCGTGCTGCGATTTTTGAGAATGGCGC CATCGCGAAAGATGTAGTCGGCCAGAGCGTTGCCTTTATCGCGAAGA AAGCAAATATCAATATACCGGAGGGTACCCGTATTCTGGTTGTTGAAG CTCGCGGCGTCGGAGCAGAGGATGTCATATGTAAGGAAAAAATGTGT CCAGTTATGTGCGCCTTAAGCTACAAGCACTTCGAGGAAGGTGTAGA AATCGCACGTACGAACTTGGCCAACGAAGGTAACGGCCATACCTGTG CGATCCATTCCAACAATCAGGCGCATATCATACTGGCAGGTTCAGAA CTGACGGTTTCGCGGATCGTGGTCAATGCGCCGAGTGCCACTACAG CAGGCGGTCACATCCAAAATGGTCTGGCAGTGACAAATACGCTCGGA TGCGGGAGTTGGGGTAATAACTCTATCTCCGAGAACTTTACTTATAAA CACCTGTTAAACATTAGCCGCATAGCGCCGCTTAATTCAAGCATTCAC ATTCCTGATGACAAAGAGATCTGGGAACTCTAA
4hbd	ATGCAACTGTTCAAACTGAAATCAGTCACACATCACTTCGATACTTTC GCGGAATTTGCCAAAGAGTTCTGTCTTGGAGAACGTGATTTAGTAATT ACCAACGAATTCATTTACGAACCGTATATGAAGGCATGTCAGTTGCCC TGCCATTTTGTTATGCAGGAGAAATATGGGCAAGGCGAGCCATCTGA CGAGATGATGAATAACATCTTGGCAGACATCCGTAATATCCAGTTTGA CCGCGTGATCGGTATTGGGGGTGGTACGGTTATTGACATCTCGAAAT TATTTGTGCTGAAAGGACTAAATGATGTGCTCGATGCGTTCGATCGCA AGATACCGCTGATTAAAGAGAAAGAACTGATCATTGTGCCCACCACAT GCGGGACGGGTAGCGAGGTGACGAATATTTCGATCGCGGAGATCAA AAGCCGTCATACCAAAATGGGTTTGGCTGACGATGCTATTGTTGCAG ACCACGCGATCATCATACCAGAGCTTCTGAAAAGCCTGCCGTTCCAT TTTTATGCATGCAGTGCAATAGATGCTCTGATCCATGCCATCGAGTCA TATGTTTCTCCTAAAGCCAGTCCATATTCTCGTCTGTTCAGTGAGGCG GCATGGGATATTATCCTGGAGGTATTCAAGAAAATAGCCGAACACGG

	CCCTGAATACCGCTTTGAGAAGCTGGGAGAAATGATCATGGCCTCCA<br>ACTATGCTGGTATAGCCTTCGGGAATGCAGGCGTGGGTGCCGTTCAC<br>GCTCTAAGCTATCCATTGGGAGGCAATTATCATGTGCCGCATGGCGA<br>GGCTAACTATCAGTTTTTTACAGAGGTCTTTAAAGTATACCAAAAGAAA<br>AATCCTTTCGGCTATATAGTCGAACTCAACTGGAAGCTGTCCAAGATT<br>CTGAACTGTCAGCCTGAATACGTCTATCCGAAACTGGATGAGTTACTC<br>GGCTGTCTTCTGACCAAAAAACCGCTGCACGAATACGGCATGAAAGA<br>TGAAGAGGTACGTGGATTTGCGGAATCAGTGCTTAAGACTCAGCAGC<br>GGTTGCTCGCGAATAATTATGTTGAGCTTACTGTTGATGAAATTGAAG<br>GTATCTACAGACGACTGTACTAA
kgd	ATGAGCAGCGCTAGTACTTTCGGCCAGAATGCGTGGCTGGTAGACGA<br>GATGTTCCAGCAGTTCCAGAAGGACCCCAAGTCCGTGGACAAGGAAT<br>GGAGAGAACTCTTTGAGGCGCAGGGGGGACCAAATACTACCCCCGC<br>TACAACAGAAGCACAGCCTTCAGCGCCCAAGGAGTCTGCGAAACCAG<br>CACCAAAGGCTGCCCCTGCAGCCAAGGCAGCACCGCGCGTAGAAAC<br>CAAGCCGGCCGACAAGACCGCCCCTAAGGCCAAGGAGTCCTCAGTG<br>CCACAGCAACCTAAGCTTCCGGAGCCAGGACAAACCCCAATCAGGG<br>GTATTTTCAAGTCCATCGCGAAGAACATGGATATCTCCCTGGAAATCC<br>CAACCGCAACCTCGGTTCGCGATATGCCAGCTCGCCTCATGTTCGAA<br>AACCGCGCGATGGTCAACGATCAGCTCAAGCGCACCCGCGGTGGCA<br>AGATCTCCTTCACCCACATCATTGGCTACGCCATGGTGAAGGCAGTC<br>ATGGCTCACCCGGACATGAACAACTCCTACGACGTCATCGACGGCAA<br>GCCAACCCTGATCGTGCCTGAGCACATCAACCTGGGCCTTGCTATCG<br>ACCTTCCTCAGAAGGACGGCTCCCGCGCACTTGTCGTAGCAGCCATC<br>AAGGAAACCGAGAAGATGAACTTCTCCGAGTTCCTCGCAGCCTACGA<br>AGACATCGTGGCACGCTCCCGCAAGGGCAAGCTCACCATGGATGAC<br>TACCAGGGCGTTACCGTTTCCTTGACCAACCCAGGTGGCATCGGTAC<br>CCGCCACTCTGTTCCACGTCTAACCAAGGGCCAGGGCACCATCATCG<br>GTGTCGGTTCCATGGATTACCCAGCAGAGTTCCAGGGCGCTTCAGAA<br>GACCGCCTTGCAGAGCTCGGCGTTGGCAAACTTGTCACCATCACCTC<br>CACCTACGATCACCGCGTGATCCAGGGTGCTGTGTCCGGTGAATTCC

	TGCGCACCATGTCTCGCCTGCTCACCGATGATTCCTTCTGGGATGAG<br>ATCTTCGACGCAATGAACGTTCCTTACACCCCAATGCGTTGGGCACA<br>GGACGTTCCAAACACCGGTGTTGATAAGAACACCCGCGTCATGCAGC<br>TCATTGAGGCATACCGCTCCCGTGGACACCTCATCGCTGACACCAAC<br>CCACTTTCATGGGTTCAGCCTGGCATGCCAGTTCCAGACCACCGCGA<br>CCTCGACATCGAGACCCACAACCTGACCATCTGGGATCTGGACCGTA<br>CCTTCAACGTCGGTGGCTTCGGCGGCAAGGAGACCATGACCCTGCG<br>CGAGGTACTGTCCCGCCTCCGCGCTGCGTACACCCTCAAGGTCGGC<br>TCCGAATACACCCACATCCTGGACCGCGACGAGCGCACCTGGCTGC<br>AGGACCGCCTCGAGGCCGGAATGCCAAAGCCAACCCAGGCAGAGCA<br>GAAGTACATCCTGCAGAAGCTGAACGCCGCGGAGGCTTTCGAGAACT<br>TCCTGCAGACCAAGTACGTCGGCCAGAAGCGCTTCTCCCTCGAAGGT<br>GCAGAAGCACTTATCCCACTGATGGACTCCGCCATCGACACCGCCGC<br>AGGCCAAGGCCTCGACGAAGTTGTCATCGGTATGCCACACCGTGGT<br>CGCCTCAACGTGCTGTTCAACATCGTGGGCAAGCCACTGGCATCCAT<br>CTTCAACGAGTTTGAAGGCCAAATGGAGCAGGGCCAGATCGGTGGC<br>TCCGGTGACGTGAAGTACCACCTCGGTTCCGAAGGCCAGCACCTGC<br>AGATGTTCGGCGACGGCGAGATCAAGGTCTCCCTGACTGCTAACCC<br>GTCCCACCTGGAAGCTGTTAACCCAGTGATGGAAGGTATCGTCCGCG<br>CAAAGCAGGACTACCTGGACAAGGGCGTAGACGGCAAGACTGTTGT<br>GCCACTGCTGCTCCACGGTGACGCTGCATTCGCAGGCCTGGGCATC<br>GTGCCAGAAACCATCAACCTGGCTAAGCTGCGTGGCTACGACGTCG<br>GCGGCACCATCCACATCGTGGTGAACAACCAGATCGGCTTCACCACC<br>ACCCCAGACTCCAGCCGCTCCATGCACTACGCAACCGACTACGCCAA<br>GGCATTCGGCTGCCCAGTCTTCCACGTCAACGGCGACGACCCAGAG<br>GCAGTTGTCTGGGTTGGCCAGCTGGCCACCGAGTACCGTCGTCGCT<br>TCGGCAAGGACGTCTTCATCGACCTCGTCTGCTACCGCCTCCGCGGC<br>CACAACGAAGCTGATGATCCTTCCATGACCCAGCCAAAGATGTATGA<br>GCTCATCACCGGCCGCGAGACCGTTCGTGCTCAGTACACCGAAGAC<br>CTGCTCGGACGTGGAGACCTCTCCAACGAAGATGCAGAAGCAGTCG<br>TCCGCGACTTCCACGACCAGATGGAATCTGTGTTCAACGAAGTCAAG<br>GAAGGCGGCAAGAAGCAGGCTGAGGCACAGACCGGCATCACCGGCT

	CCCAGAAGCTTCCACACGGCCTTGAGACCAACATCTCCCGTGAAGAG<br>CTCCTGGAACTGGGACAGGCTTTCGCCAACACCCCAGAAGGCTTCAA<br>CTACCACCCACGTGTGGCTCCCGTTGCTAAGAAGCGCGTCTCCTCTG<br>TCACCGAAGGTGGCATCGACTGGGCATGGGGCGAGCTCCTCGCCTT<br>CGGTTCCCTGGCTAACTCCGGCCGCTTGGTTCGCCTTGCAGGTGAAG<br>ATTCCCGCCGCGGTACCTTCACCCAGCGCCACGCAGTTGCCATCGAC<br>CCAGCGACCGCTGAAGAGTTCAACCCACTCCACGAGCTTGCACAGTC<br>CAAGGGCAACAACGGTAAGTTCCTGGTCTACAACTCCGCACTGACCG<br>AGTACGCAGGCATGGGCTTCGAGTACGGCTACTCCGTAGGAAACGA<br>AGACTCCATCGTTGCATGGGAAGCACAGTTCGGCGACTTCGCCAACG<br>GCGCTCAGACCATCATCGATGAGTACGTCTCCTCAGGCGAAGCTAAG<br>TGGGGCCAGACCTCCAAGCTGATCCTTCTGCTGCCTCACGGCTACGA<br>AGGCCAGGGCCCAGACCACTCTTCCGCACGTATCGAGCGCTTCCTG<br>CAGCTGTGCGCTGAGGGTTCCATGACTGTTGCTCAGCCATCCACCCC<br>AGCAAACCACTTCCACCTACTGCGTCGTCACGCTCTGTCCGACCTGA<br>AGCGTCCACTGGTTATCTTCACCCCGAAGTCCATGCTGCGTAACAAG<br>GCTGCTGCCTCCGCACCAGAAGACTTCACTGAGGTCACCAAGTTCCA<br>GTCCGTGATCAACGATCCAAACGTTGCAGATGCAGCCAAGGTGAAGA<br>AGGTCATGCTGGTCTCCGGCAAGCTGTACTACGAATTGGCAAAGCGC<br>AAGGAGAAGGACGGACGCGACGACATCGCGATCGTTCGTATCGAAA<br>TGCTCCACCCAATTCCGTTCAACCGCATCTCCGAGGCTCTTGCCGGC<br>TACCCTAACGCTGAGGAAGTCCTCTTCGTTCAGGATGAGCCAGCAAA<br>CCAGGGCCCATGGCCGTTCTACCAGGAGCACCTCCCAGAGCTGATC<br>CCGAACATGCCAAAGATGCGCCGCGTTTCCCGCCGCGCTCAGTCCT<br>CCACCGCAACTGGTGTTGCCAAGGTGCACCAGCTGGAGGAGAAGCA<br>GCTTATCGACGAGGCTTTCGAGGCTTAA
bld_M	ATGATTAAAGACACGCTAGTTTCTATAACAAAAGATTTAAAATTAAAAA<br>CAAATGTTGAAAATGCCAATCTAAAGAACTACAAGGATGATTCTTCAT<br>GTTTCGGAGTTTTCGAAAATGTTGAAAATGCTATAAGCAATGCCGTAC<br>ACGCACAAAAGATATTATCCCTTCATTATACAAAAGAACAAAGAGAAA<br>AAATCATAACTGAGATAAGAAAGGCCGCATTAGAAAATAAAGAGATTC

	TAGCTACAATGATTCTTGAAGAAACACATATGGGAAGATATGAAGATA<br>AAATATTAAAGCATGAATTAGTAGCTAAATACACTCCTGGGACAGAAG<br>ATTTAACTACTACTGCTTGGTCAGGAGATAACGGGCTTACAGTTGTAG<br>AAATGTCTCCATATGGCGTTATAGGTGCAATAACTCCTTCTACGAATC<br>CAACTGAAACTGTAATATGTAATAGTATAGGCATGATAGCTGCTGGAA<br>ATACTGTGGTATTTAACGGACATCCAGGCGCTAAAAAATGTGTTGCTT<br>TTGCTGTCGAAATGATAAATAAAGCTATTATTTCATGTGGTGGTCCTG<br>AGAATTTAGTAACAACTATAAAAAATCCAACTATGGACTCTCTAGATGC<br>AATTATTAAGCACCCTTCAATAAAACTACTTTGCGGAACTGGAGGGCC<br>AGGACTCGTAAAAACCCTCTTAAATTCTGGTAAGAAAGCTATAGGTGC<br>TGGTGCTGGAAATCCACCAGTTATTGTAGATGATACTGCTGATATAGA<br>AAAGGCTGGTAAGAGTATCATTGAAGGCTGTTCTTTTGATAATAATAT<br>CCCTTGTATTGCAGAAAAAGAAGTATTTGTTTTTGAGAACGTTGCAGA<br>TGATTTAATATCTAACATGCTAAAAAATAATGCTGTAATTATAAATGAA<br>GATCAAGTATCAAAGTTAATAGATTTAGTATTACAAAAAAATAATGAAA<br>CTCAAGAATACTCTATAAATAAGAAATGGGTCGGAAAAGATGCAAAAT<br>TATTCTTAGATGAAATAGATGTTGAGTCTCCTTCAAGTGTTAAATGCAT<br>AATCTGCGAAGTAAGTGCAAGGCATCCATTTGTTATGACAGAACTCAT<br>GATGCCAATATTACCAATTGTAAGAGTTAAAGATATAGATGAAGCTATT<br>GAATATGCAAAAATAGCAGAACAAAATAGAAAACATAGTGCCTATATT<br>TATTCAAAAAATATAGACAACCTAAATAGGTTTGAAAGAGAAATCGATA<br>CTACTATCTTTGTAAAGAATGCTAAATCTTTTGCCGGTGTTGGTTATGA<br>AGCAGAAGGCTTTACAACTTTCACTATTGCTGGATCCACTGGTGAAG<br>GAATAACTTCTGCAAGAAATTTTACAAGACAAAGAAGATGTGTACTCG<br>CCGGTTAA
cat2	ATGAAAGACGTGTTAGCGGAATATGCCTCCCGAATTGTTTCGGCCGA<br>AGAGGCAGTCAAACATATCAAAAATGGAGAGCGTGTCGCTTTATCAC<br>ATGCTGCCGGAGTTCCTCAGAGTTGTGTTGACGCACTGGTGCAACAG<br>GCGGACCTGTTTCAGAATGTGGAGATTTACCACATGCTGTGTCTCGG<br>CGAAGGAAAATATATGGCACCTGAAATGGCCCCTCACTTCCGGCACA<br>TAACCAATTTTGTTGGTGGTAACTCTCGTAAAGCAGTGGAGGAAAATA

	GAGCCGACTTCATTCCGGTATTCTTTTATGAAGTGCCATCAATGATTC<br>GGAAAGATATCCTTCATATAGATGTGGCCATTGTCCAACTCTCAATGC<br>CAGATGAGAATGGTTACTGCAGCTTTGGCGTATCTTGCGATTATAGCA<br>AACCGGCGGCGGAATCGGCGCATTTAGTTATTGGGGAAATCAACCGT<br>CAGATGCCATATGTGCATGGTGACAACTTGATTCACATATCGAAGTTG<br>GATTACATCGTGATGGCGGATTACCCAATTTATTCTCTGGCGAAGCCC<br>AAAATCGGAGAAGTAGAGGAAGCTATCGGCCGTAACTGTGCCGAGCT<br>TATTGAAGATGGTGCCACCCTACAGCTGGGTATCGGCGCGATTCCGG<br>ATGCAGCTCTGCTGTTTCTGAAGGACAAAAAAGATCTGGGGATTCATA<br>CTGAAATGTTCTCCGATGGCGTTGTTGAACTGGTGCGCAGTGGTGTA<br>ATTACTGGAAAAAAAAAGACATTGCATCCCGGTAAGATGGTCGCGAC<br>GTTTCTTATGGGATCAGAAGACGTGTATCATTTCATCGACAAGAATCC<br>GGATGTGGAACTGTATCCGGTTGATTACGTCAATGATCCGAGGGTTA<br>TCGCTCAGAATGATAATATGGTCAGCATCAATAGCTGTATCGAGATCG<br>ATCTAATGGGCCAAGTGGTGAGCGAGTGCATAGGCTCCAAACAGTTT<br>AGTGGCACCGGGGGTCAAGTAGATTATGTCCGCGGGGCAGCTTGGT<br>CTAAAAACGGCAAAAGCATCATGGCAATTCCCTCAACAGCCAAAAAC<br>GGTACTGCATCTCGGATAGTTCCTATAATTGCAGAGGGCGCTGCTGT<br>AACAACCCTCCGCAACGAAGTCGACTACGTTGTTACGGAATATGGGA<br>TAGCACAGTTAAAAGGTAAGAGTTTGCGTCAGCGCGCAGAAGCTCTT<br>ATTGCGATAGCCCACCCGGACTTTAGAGAGGAACTGACGAAGCATCT<br>GCGCAAACGTTTTGGTTAA

Supplementary Table 4. Promoter sequences of 1,4-BDO biosynthetic pathway genes

Gene/ Pathway	Promoter	Sequence (5` → 3`)
ald	P1+U2	TTTACAGCTAGCTCAGTCCTAGGTATTATGCTAGCTTTGGAATTC ATTAAAGAGGAGAAAGGTACC
4hbd	P7+U5	TAATTCCTAATTTTTGTTGACACTCTATCGTTGATAGAGTTATTTT ACCACTCCCTATCAGTGATAGAGAAAATTTGAAAGAGGAGAAAT TAAGC
cat2	P6+U3	TTGACAATTAATCATCCGGCTCGTATAATGTGTGGAATTGTGAGT TTGATTAAAGAGGAGAAATTAAGC
sucD	P3+U9	ATTCCACTAATTTATTCCATGTCACACTTTTCGCATCTTTGTTATG CTATGGTTATTTCATACCATAATTTGGAATTCAAAAGATCTTTTAA GAAGGAGATATACAT
upstream	pTac	TTGACAATTAATCATCGGCTCGTATAATGTGTGGAATTGTGAGC GGATAACAATTTCACACAGGAAACAGAATTC
downstream	pTrc	TTGACAATTAATCATCCGGCTCGTATAATGTGTGGAATTGTGAGC GGATAACAATTTCACACAGGAAACAGACC

Supplementary Table 5. Terminator sequences of 1,4-BDO biosynthetic pathway genes.

Gene/ pathway	Terminator	Sequence (5` → 3`)
ald	rpoC	GTGTAGAACCAACACGTGTGGTAATCGTTAATCCGCAAATAACGTAAAAACCCGCTTCGGCGGGTTTTTTTATGGGGGGAGTTTAGGGAAAGAGCATTTGTCACCACAGACGTCTAGTTGGACCGGAATGGCTACTTAGGCTGCATGATGGAC
4hbd	ilv GEDA	TCCGGAATCTCATTGGTTGTTAGAGATCAAGCCTTAACGAACTAAGACCCCCGCACCGAAAGGTCCGGGGGTTTTTTTTGACCTTAAAAACATAACCGAGGAGCAGACACCAATCCAGCCTGACTCGTCAGGTTCAAGAATGACAGGTCGTAGCGTAAC
cat2	his[min]	AGCGTCTGAACACGAAGATTGGGAACTGCCAGGCATCAAATAAAACGAAAGGCTCAGTCGAAAGACTGGGCCTTTCGTTTTATCTGTTGTTTGTCGGTGAACGCTCTCCTGTAGACCACCGTCTTGCACAGCCATCCTATGATCGAGTTGTAACGCGTGAA
sucD	M13 central	CTTGACGTTCTGTGGTCTGAGGGAACTGCCAGGCATCAAATAAAACGAAAGGCTCAGTCGGAAGACTGGGCCTTTCGTTTTATCTGTTGTTTGTCGGTGAACGCTCTCCTGGCGATACTGACTTGAGCAATTGGTCGATCATCTAAGCAGCTGGATTGCTC
upstream / downstream	rrnB	CTTGGCTGTTTTGGCGGATGAGAGAAGATTTTCAGCCTGATACAGATTAAATCAGAACGCAGAAGCGGTCTGATAAAACAGAATTTGCCTGGCGGCAGTAGCGCGGTGGTCCCACCTGACCCCATGCCGAACTCAGAAGTGAAACGCCGTAGCGCCGATGGTAGTGTGGGGTCTCCCCATGCGAGAGTAGGGAACTGCCAGGCATCAAATAAAACGAAAGGCTCAGTCGAAAGACTGGGCCTTTCGTTTTATCTGTTGTTTGTCGGTGAACGCTCTCCTGAGTAGGACAAATCCGCCGGGAGCGGATTTGAACGTTGCGAAGCAACGGCCCGGAGGGTGGCGGGCAGGACGCCCGCCATAAACTGCCAGGCATCAAATTAAGCAGAAGGCCATCCTGACGGATGGCCTTTTTGCGTTTCTACAAACTCTT

Supplementary Table 6. Oligonucleotides for preparation of DNA substrates to construct YSB20.

Gene	Name	Sequence (5` → 3`)
ald	P1+U2 fwd	catcgtttttgccaaatgtaacgggcaggttgtctggcttaagcattgttTTTACAGCTAGCTCAGTCCTAG
	ald fwd	GAATTCATTAAAGAGGAGAAAGGTACCATGAATAAAGACACACTGATCCCT
	ald rev	CACACGTGTTGGTTCTACACTTAGCCGGCCAGTACACAG
	rpoC rev	ctgaacccagaaaggctcaggccgataagaatggggagcaatttcttcatGTCCATCATGCAGCCTAAGT
4hbd	P7+U5 fwd	ccacagacgtctagttggaccggaatggctacttaggctgcatgatggacTAATTCCTAATTTTTGTTGACACTCTATC
	4hbd fwd	GAGAAAATTTGAAAGAGGAGAAATTAAGCATGCAACTTTTCAAACTCAAGAGT
	4hbd rev	ACAACCAATGAGATTCCGGATTAGTAGAGTCTTCTGTAGATACCTTCG
	ilv GEDA rev	CAATTCCCCTATAGTGAGTCGTATTAGTTACGCTACGACCTGTCAT
cat2	P6+U3 fwd	ccaatccagcctgactcgtcaggttcaagaatgacaggtcgtagcgtaacTTGACAATTAATCATCCGGCTC
	cat2 fwd	GAGTTTGATTAAAGAGGAGAAATTAAGCATGCAATGGCAAGAACTTTACC
	cat2 rev	AATCTTCGTGTTCAGACGCTTTATTCGAAGCGTCGGCG
	his[min] rev	ctgaacccagaaaggctcaggccgataagaatggggagcaatttcttcatTTCACGCGTTACAACTCGAT

sucD	P3+U9 fwd	tagaccaccgtcttgcacagccatcctatgatcgagttgtaacgcgtgaaATTCCACTAATTTATTCCATGTCACAC
	sucD fwd	TCAAAAGATCTTTTAAGAAGGAGATATACATATGGAAATCAAAGAAATGGTGAGC
	sucD rev	TCAGACCACAGAACGTCAAGTTAGAGTTCCCAGATTTCTTTGTCATC
	M13 central rev	CAATTCCCCTATAGTGAGTCGTATTAGAGCAATCCAGCTGCTTAGA
4hbd/sucD	T7 tolC	TAATACGACTCACTATAGGGGAATTGTGAGCGTTTAACTTTAAGAAGGAGATATACATatgaagaaattgctccccattcttatcggcctgagcctttctgggttcag
	tolC ON rev	CTGAACCCAGAAAGGCTCA

* Homology arm sequences are written in blue lowercase letters. Green letters represent the *T7* promoter and purple letters represent RBS.

Supplementary Table 7. Oligonucleotides for preparation of DNA substrates to construct YSB27.

Gene	Name	Sequence (5` → 3`)
YSB_Up_1st	YSB_Up_1st fwd	catcgtttttgccaaatgtaacgggcaggttgtctggcttaagcattgttTTGACAATTAATCATCGGCT
	YSB_Up_1st rev	cgaactgaacccagaaaggctcaggccgataagaatggggagcaatttctCTTTGGATTTCCCCTGATTC
YSB_Up_2nd	YSB_Up_2nd fwd	catgggcgtatatgaacataaagtggccaagaatcaggggaaatccaaagGCGTCTGGTACAATTTGCAC
	YSB_Up_2nd rev	CCCTATAGTGAGTCGTATTAGTTCGACTATATAGCCGAAA
YSB_Up_3rd	YSB_Up_3rd fwd	ggtctttaaagtataccaaaagaaaaatcctttcggctatatagtcgaacTCAACTGGAAGCTGTCCAAG
	YSB_Up_3rd rev	cgaactgaacccagaaaggctcaggccgataagaatggggagcaatttctCCGCCGACGTCGTAGCCACG
YSB_Up_4th	YSB_Up_4th fwd	gtgatccagggtgctgtgtccggtgaattcctgcgcaccatgtctcgcctGCTCACCGATGATTCCTTCT
	YSB_Up_4th rev	CCCTATAGTGAGTCGTATTAACCGAAGGCGAGGAGCTCGC
YSB_Up_5th	YSB_Up_5th fwd	tcaccgaaggtggcatcgactgggcatggggcgagctcctcgccttcggtTCCCTGGCTAACTCCGGCCG
	YSB_Up_5th rev	cgaactgaacccagaaaggctcaggccgataagaatggggagcaatttctAAGAGTTTGTAGAAACGCAA
YSB_Down_1st	YSB_Down_1st fwd	agcagaaggccatcctgacggatggcctttttgcgtttctacaaactcttTTGACAATTAATCATCCGGCTC
	YSB_Down	CAATTCCCCTATAGTGAGTCGTATTATTAACCGGCGA

	_1st rev	GTACACATC
YSB_Down_2nd	YSB_Down_2nd fwd	cttctgcaagaaattttacaagacaaa
	YSB_Down_2nd rev	cgaactgaacccagaaaggctcaggccgataagaatggggagcaatttct AAGAGTTTGTAGAAACGCAA
	T7_tolC	TAATACGACTCACTATAGGGGAATTGTGAGCGTTTAA CTTTAAGAAGGAGATATACATatgaagaaattgctccccattctt atcggcctgagcctttctgggttcag
	tolC ON rev	CTGAACCCAGAAAGGCTCA

* Homology arm sequences are written in blue lowercase letters. Green letters represent the *T7* promoter and purple letters represent RBS.

Supplementary Table 8. Oligonucleotides for preparation of DNA substrates to construct W029-11.

Gene	Name	Sequence (5` → 3`)
W029_Up_1st	W029_Up_1st fwd	catcgtttttgccaaatgtaacgggcaggttgtctggcttaagcattgttTTGACAATTAATCATCGGCT
	W029_Up_1st rev	cgaactgaacccagaaaggctcaggccgataagaatggggagcaatttctCTTTGGATTTCCCCTGATTC
W029_Up_2nd	W029_Up_2nd fwd	catgggcgtatatgaacataaagtggccaagaatcaggggaaatccaaagGCGTCTGGTACAATTTGCAC
	W029_Up_2nd rev	CCCTATAGTGAGTCGTATTATATGATGATCGCGTGGTCTG
W029_Up_3rd	W029_Up_3rd fwd	aaatgggtttggctgacgatgctattgttgcagaccacgcgatcatcataCCAGAGCTTCTGAAAAGCCT
	W029_Up_3rd rev	cgaactgaacccagaaaggctcaggccgataagaatggggagcaatttctAGGCGAGACATGGTGCGCAG
W029_Up_4th	W029_Up_4th fwd	gtgatccagggtgctgtgtccggtgaattcctgcgcaccatgtctcgcctGCTCACCGATGATTCCTTCT
	W029_Up_4th rev	ACAATTCCCCTATAGTGAGTCGTATTACCAGGTGGGACGGGTTAG
W029_Up_5th	W029_Up_5th fwd	cggcgacggcgagatcaaggtctccctgactgctaacccgtcccACCTGGAAGCTGTTAACCCAGTGATGGA
	W029_Up_5th rev	cgaactgaacccagaaaggctcaggccgataagaatggggagcaatttctACCGAAGGCGAGGAGC
	W029_Up_6th fwd	tcaccgaaggtggcatcgactgggcatggggcgagctcctcgccttcggtTCCCTGGCTAACTCCGGCCG
	W029_Up_	CTCCTTCTTAAAGTTAAACGCTCACAATTCCCCTATA

	6th rev	GTGAGTCGTATTACTTTGCCAATTCGTAGTACAGC
	W029_Up_ 7th fwd	aaggtgaagaaggtcatgctggtctccggcaagctgtactacgaattggcaa agCGCAAGGAGAAGGAC
	W029_Up_ 7th rev	cgaactgaacccagaaaggctcaggccgataagaatggggagcaatttct AAGAGTTTGTAGAAACGCAA
W029_Down_1st	W029_Dow n_1st fwd	agcagaaggccatcctgacggatggcctttttgcgtttctacaaactcttTTG ACAATTAATCATCCGGCT
	W029_Dow n_1st rev	CTCCTTCTTAAAGTTAAACGCTCACAATTCCCCTATA GTGAGTCGTATTACTAAATCTATTAACTTTGATACTTG ATCTTCATTTATAATTA
W029_Down_2nd	W029_Dow n_2nd fwd	taatgctgtaattataaatgaagatcaagtatcaaagttaatagatttagTATT ACAAAAAAATAATGAAACTCAAGAATACTC
	W029_Dow n_2nd rev	cgaactgaacccagaaaggctcaggccgataagaatggggagcaatttct TTCAGAAACAGCAGAGCTGC
W029_Down_3rd	W029_Dow n_3rd fwd	ctacagctgggtatcggcgcgattccggatgcagctctgctgtttctgaaGGA CAAAAAAGATCTGGGGATTC
	W029_Dow n_3rd rev	CTCCTTCTTAAAGTTAAACGCTCACAATTCCCCTATA GTGAGTCGTATTAGTGCTATCCCATATTCCGTAACA
W029_Down_4th	W029_Dow n_4th fwd	aaccctccgcaacgaagtcgactacgttgttacggaatatgggatagcacA GTTAAAAGGTAAGAGTTTGCGTC
	W029_Dow n_4th rev	cgaactgaacccagaaaggctcaggccgataagaatggggagcaatttct AAGAGTTTGTAGAAACGCAAAAAGG
	T7_tolC	TAATACGACTCACTATAGGGGAATTGTGAGCGTTTAA CTTTAAGAAGGAGATATACATatgaagaaattgctccccattctt atcggcctgagcctttctgggttcag
	tolC ON rev	CTGAACCCAGAAAGGCTCA

* Homology arm sequences are written in blue lowercase letters. Green letters represent the

T7 promoter and purple letters represent RBS.

Supplementary Table 9. Oligonucleotides for colony PCR.

Gene	Name	Sequence (5` → 3`)
ald	ald col fwd	TCAAAGGGTAGCAAGACTGC
4hbd	4hbd col fwd	ATGATGCCAATATTGCCAATTGT
cat2	cat2 col fwd	CTCGGATGCCTTCTTACCAA
sucD	sucD col fwd	AGTGGATCCGGAGGTCAA
Universal rev	tolC col rev	GCGTGCTTGCTGATAAACT

Gene	Name	Sequence (5` → 3`)
YSB_Up_1st	YSB_Up_1st col fwd	TCAAAGGGTAGCAAGACTGC
YSB_Up_2nd	YSB_Up_2nd col fwd	CACACAGGAAACAGAATTCATGG
YSB_Up_3rd	YSB_Up_3rd col fwd	GGGATATTATCCTGGAGGTATTCAA
YSB_Up_4th	YSB_Up_4th col fwd	AAACTTGTCACCATCACCTCC
YSB_Up_5th	YSB_Up_5th col fwd	TGGAACTGGGACAGGCTTT
YSB_Down_1st	YSB_Down_1st col fwd	TTCGTTTTATCTGTTGTTTGTCGG
YSB_Down_2nd	YSB_Down_2nd col fwd	GAAGCAGAAGGCTTTACAACTTT
Universal rev	tolC col rev	GCGTGCTTGCTGATAAACT

Gene	Name	Sequence (5` → 3`)
W029_Up_1st	W029_Up_1st col fwd	TCAAAGGGTAGCAAGACTGC

W029_Up_2nd	W029_Up_2nd col fwd	ACAGGAAACAGAATTCATGGAAATAA
W029_Up_3rd	W029_Up_3rd col fwd	AAAATGGGTTTGGCTGACGAT
W029_Up_4th	W029_Up_4th col fwd	AAACTTGTCACCATCACCTCC
W029_Up_5th	W029_Up_5th col fwd	TTCAACGAGTTTGAAGGCCA
W029_Up_6th	W029_Up_6th col fwd	TGGAACTGGGACAGGCTTT
W029_Up_7th	W029_Up_7th col fwd	ACTGGTTATCTTCACCCCGA
W029_Down_1st	W029_Down_1st col fwd	AAAGGCTCAGTCGAAAGACTG
W029_Down_2nd	W029_Down_2nd col fwd	CCACCAGTTATTGTAGATGATACTG
W029_Down_3rd	W029_Down_3rd col fwd	TGTGCATGGTGACAACTTGA
W029_Down_4rd	W029_Down_4th col fwd	GGGGCAGCTTGGTCTAAAA
Universal rev	tolC col rev	GCGTGCTTGCTGATAAACT

Supplementary Table 10. Oligonucleotides for construction of engineered 1,4-BDO biosynthetic pathway plasmids

Gene	Name	Sequence (5` → 3`)
sucCD	sucCD fwd	gctagaattcATGAACTTACATGAATATCAGG
	sucCD rev	gcaaggtaccTTATTTCAGAACAGTTTTCAG
sucD	sucD fwd	GAAAACTGTTCTGAAATAAGTCACACAGGAAACAGAATTCATGGAAATAAAAGAGATG
	sucD rev	TTCAGTTTGAACAGTTGCATGAATTCTGTTTCCTGTGTGATTAGAGTTCCCAGATCTC
4hbd	4hbd fwd	AAGAGATCTGGGAACTCTAATCACACAGGAAACAGAATTCATGCAACTGTTCAAACTG
	4hbd rev	CGACTCTAGAGGATCCCCGGTTAGTACAGTCGTCTGTAGATACCTTC
kgd	kgd fwd	CTAACCGGGGATCCTCTAGTCACACAGGAAACAGAATTCAtgagcagcgctagtacttt
	kgd rev	TTGCATGCCTGCAGGTCGACtctagttaagcctcgaaagcctcgtc
cat2	cat2 fwd	gctaGAATTCATGAAAGACGTGTTAGCGGA
	cat2 rev	gcaaAAGCTTTTAACCAAAACGTTTGCGCA
bld_M	bld_M fwd	AGGAAACAGACCATGATTAAAGACACGCTAGT
	bld_M rev	CATCTGTTTCGAATTCTTAACCGGCGAGTACACATCT
	bld_M M227L fwd	GGAACTGGAGGGCCAGGACTCGTAAAAACCCTCTTA
	bld_M M227L rev	TAAGAGGGTTTTTACGAGTCCTGGCCCTCCAGTTCC
	bld_M L273I fwd	GTTCTTTTGATAATAATATCCCTTGTATTGCAGAAA
	bld_M L273I rev	TTTCTGCAATACAAGGGATATTATTATCAAAAGAAC

Supplementary Table 11. Oligonucleotides for development of W029.

Name	Sequences (5` → 3`)
gabDF	ATGAAACTTAACGACAGTAACTTATTCCGCCAGCAGGCGTTGATTAACGG TAGGTGACACTATAGAACGCG
gabDR	TTAAAGACCGATGCACATATATTTGATTTCTAAGTAATCTTCGATGCCATTA GTGGATCTGATGGGTACC
sadF	ATGACCATTACTCCGGCAACTCATGCAATTTCGATAAATCCTGCCACGGG TAGGTGACACTATAGAACGCG
sadR	TCAGATCCGGTCTTTCCACACCGTCTGGATATTACAGAATTCGTGTAAGCT AGTGGATCTGATGGGTACC
puuCF	ATGAATTTTCATCATCTGGCTTACTGGCAGGATAAAGCGTTAAGTCTCGCT AGGTGACACTATAGAACGCG
puuCR	TCAGGCCTCCAGGCTTATCCAGATGGTTTTCAGTTCGGTGAATTTTTCAAT AGTGGATCTGATGGGTACC
RPacsF	gacattgctcgcccctatgtgtaacaaataaccacactgtgaatgttgtcTAGGTGACACTATAGAA CGCG
RPacsR	caacggtctgcgatgttggcaggaatggtgtgtttgtgaatttggctcatGGTCTGTTTCCTGTGTG AAAT
RPppcF	CCATAAGTTACGCTTATTTAAAGCGTCGTGAATTTAATGGCGTAAATTCCT AGGTGACACTATAGAACGCG
RPppcR	TTGCCGAGCATACTGACATTACTACGCAATGCGGAATATTGTTCGTTCATG GTCTGTTTCCTGTGTGAAAT

Supplementary References

1. Datsenko, K.A. & Wanner, B.L. One-step inactivation of chromosomal genes in Escherichia coli K-12 using PCR products. *Proc Natl Acad Sci U S A* **97**, 6640-6645 (2000).
2. Yuan, L.Z., Rouviere, P.E., Larossa, R.A. & Suh, W. Chromosomal promoter replacement of the isoprenoid pathway for enhancing carotenoid production in E. coli. *Metab Eng* **8**, 79-90 (2006).
3. Lee, K.H., Park, J.H., Kim, T.Y., Kim, H.U. & Lee, S.Y. Systems metabolic engineering of Escherichia coli for L-threonine production. *Mol Syst Biol* **3**, 149 (2007).
4. Palmeros, B. et al. A family of removable cassettes designed to obtain antibiotic-resistance-free genomic modifications of Escherichia coli and other bacteria. *Gene* **247**, 255-264 (2000).
5. Wong, Q.N. et al. Efficient and seamless DNA recombineering using a thymidylate synthase A selection system in Escherichia coli. *Nucleic Acids Res* **33**, e59 (2005).
